# AhR/IL-22 pathway as new target for the treatment of post-infectious irritable bowel syndrome symptoms

**DOI:** 10.1080/19490976.2021.2022997

**Published:** 2022-01-28

**Authors:** Maëva Meynier, Elodie Baudu, Nathalie Rolhion, Manon Defaye, Marjolène Straube, Valentine Daugey, Morgane Modoux, Ivan Wawrzyniak, Frédéric Delbac, Romain Villéger, Mathieu Méleine, Esther Borras Nogues, Catherine Godfraind, Nicolas Barnich, Denis Ardid, Philippe Poirier, Harry Sokol, Jean-Marc Chatel, Philippe Langella, Valérie Livrelli, Mathilde Bonnet, Frédéric Antonio Carvalho

**Affiliations:** a M2iSH, UMR 1071 INSERM, University of Clermont Auvergne, INRAE USC 2018, Clermont-Ferrand 63001, France; b NeuroDol, UMR 1107 INSERM, University of Clermont Auvergne, Clermont-Ferrand 63001, France; c Sorbonne University, INSERM, Centre de Recherche Saint-Antoine, CRSA, AP-HP, Saint Antoine Hospital, Gastroenterology Department, F-75012 Paris, France; d Paris Centre for Microbiome Medicine FHU, Paris, France; e Department of Physiology and Pharmacology, Inflammation Research Network, Snyder Institute for Chronic Diseases, Cumming School of Medicine, University of Calgary, Calgary, AB, T2N 4N1, Canada; f LMGE, CNRS 6023, University of Clermont Auvergne, Clermont-Ferrand 63001, France; g Université Paris-Saclay, Institut National de la Recherche Agronomique et Environnementale (INRAE), AgroParisTech UMR 1319 MICALIS, Jouy-en-Josas, France; h CHU Clermont-Ferrand, Neuropathology Unit, Clermont-Ferrand, France; i CHU Clermont-Ferrand, Laboratoire de Parasitologie et de Mycologie, Clermont-Ferrand, France

**Keywords:** Post-infectious ibs, colonic-associated microbiota, *citrobacter rodentium*, tryptophan, ahr, il-22, *lactococcus lactis*, well-being disorders

## Abstract

Alterations in brain/gut/microbiota axis are linked to Irritable Bowel Syndrome (IBS) physiopathology. Upon gastrointestinal infection, chronic abdominal pain and anxio-depressive comorbidities may persist despite pathogen clearance leading to Post-Infectious IBS (PI-IBS). This study assesses the influence of tryptophan metabolism, and particularly the microbiota-induced AhR expression, on intestinal homeostasis disturbance following gastroenteritis resolution, and evaluates the efficacy of IL-22 cytokine vectorization on PI-IBS symptoms. The *Citrobacter rodentium* infection model in C57BL6/J mice was used to mimic Enterobacteria gastroenteritis. Intestinal homeostasis was evaluated as low-grade inflammation, permeability, mucosa-associated microbiota composition, and colonic sensitivity. Cognitive performances and emotional state of animals were assessed using several tests. Tryptophan metabolism was analyzed by targeted metabolomics. AhR activity was evaluated using a luciferase reporter assay method. One *Lactococcus lactis* strain carrying an eukaryotic expression plasmid for murine IL-22 (*L. lactis*^IL−22^) was used to induce IL-22 production in mouse colonic mucosa. *C. rodentium*-infected mice exhibited persistent colonic hypersensitivity and cognitive impairments and anxiety-like behaviors after pathogen clearance. These post-infectious disorders were associated with low-grade inflammation, increased intestinal permeability, decrease of *Lactobacillaceae* abundance associated with the colonic layer, and increase of short-chain fatty acids (SCFAs). During post-infection period, the indole pathway and AhR activity were decreased due to a reduction of tryptophol production. Treatment with *L. lactis*^IL−22^ restored gut permeability and normalized colonic sensitivity, restored cognitive performances and decreased anxiety-like behaviors. Data from the video-tracking system suggested an upgrade of welfare for mice receiving the *L.lactis*^IL−22^ strain. Our findings revealed that AhR/IL-22 signaling pathway is altered in a preclinical PI-IBS model. IL-22 delivering alleviate PI-IBS symptoms as colonic hypersensitivity, cognitive impairments, and anxiety-like behaviors by acting on intestinal mucosa integrity. Thus, therapeutic strategies targeting this pathway could be developed to treat IBS patients suffering from chronic abdominal pain and associated well-being disorders.

## Introduction

Irritable bowel syndrome (IBS) is the most common disorder of the gut-brain interactions diagnosed in gastroenterology.^1^ Its etiology is multifactorial and its physiopathology is complex, involving colonic hypersensitivity (CHS), epithelial dysfunction, low-grade mucosal inflammation, and changes in gut microbiota composition.^2^ Acute infections of the digestive tract are a well-known risk factor leading to post-infection IBS (PI-IBS) in a subset of patients experiencing abdominal pain and transit disorders despite pathogen clearance.^3^ A large proportion of these infections involved *Enterobacteriaceae* such as Enterohaemorrhagic (EHEC) or Enteropathogen (EPEC) *Escherichia coli*.^4^ More broadly, dysbiotic fecal microbiota is reported in IBS patients with an increased relative abundance of pro-inflammatory bacteria from *Enterobacteriaceae* family and a decrease of the *Lactobacillaceae* and *Bifidobacterium* populations.^5^ Proliferation of specific bacterial species is even associated with IBS symptoms as illustrated by an increase of sulfate-reducing bacteria in patients with CHS and microbiota transfer to germ-free rats enhances sensitivity to colorectal distension suggesting a contribution of microbiota alterations in pain processing from IBS patients.^6^ Among the bacterial effectors, tryptophan derivatives could significantly participate in the IBS physiopathology. Beneficial effects of certain species are also well established. For instance, *Faecalibacterium prauznitsii* plays an important role in maintaining intestinal homeostasis by production of short-chain fatty acids (SCFAs) from dietary fibers.^7^ This species has been described as reduced in IBS patients and, in addition to exert anti-inflammatory properties,^8^ is able to reduce CHS in murine model of IBS.^9^

Gut microbiota plays an essential role in the catabolism of dietary tryptophan (Trp) into several metabolites, which modulate mucosal immunity and homeostasis, intestinal motility and neurobiological functions.^10–12^ Trp metabolism follows three pathways: the serotonin pathway in enterochromaffin cells, the kynurenine pathway in epithelial and immune cells, and the indole pathway in the gut microbiota.^10^ A wide range of bacterial species are able to produce indole including *Lactobacillus, Clostridium*, and *Bacteroides* spp.^13^ Indole derivatives are enabled to bind and activate aryl hydrocarbon receptor (AhR), inducing expression of downstream cytokines as interleukin-22 (IL-22)^14^ by innate lymphoid cells 3 (ILC3)^15^ and thereby regulating the epithelial integrity and immunity.^16^ Mice deficient for AhR or IL-22 expression have been shown to succumb to *C. rodentium* infection.^15,17^ Remarkably, gut microbiota composition can be altered by an AhR ligand-free diet or in *il-22* knock-out mice.^18,19^ Recently, microbiota-induced expression of AhR in colonic neurons has been shown to be important in neuronal programming by microbiota to maintain gut motility and thus to regulate intestinal physiology.^11^ Finally, dysregulation in multiple pathways of Trp metabolism may be involved in IBS.^1^

Hence, aims of this study were (i) to determine the role of AhR/IL-22 pathway on PI-IBS physiopathology and (ii) to characterize a potential therapeutic target for this gastrointestinal disorder. For these purposes, we have validated *C. rodentium* infection mouse model as a model of PI-IBS characterizing intestinal and behavioral disturbances after clearance of the pathogen.

## Results

### Citrobacter rodentium *infection induced long-lasting disturbances of colonic mucosa homeostasis*

**Figure 1.**
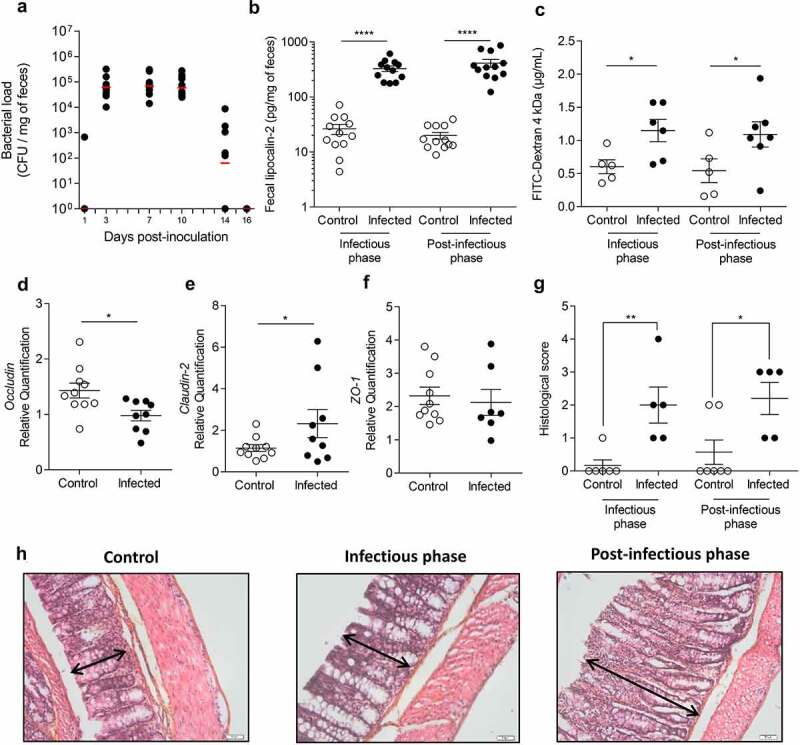
Transient *C. rodentium* infection induces persistent perturbations in mice.

At 3 days post-infection (DPI), all mice were colonized with *C. rodentium* (Figure 1A). Bacterial load rapidly increased reaching a plateau between 3 and 10 DPI, defining the peak of infection. At 16 DPI, *C. rodentium* was no longer detected by microbiological approaches in fecal samples, defining the beginning of post-infectious period. At 7 DPI, fecal lipocalin-2 level (colonic low-grade inflammation marker) in infected mice was significantly higher than in control group (p < 0.0001) (Figure 1B). This difference persisted up to 23–24 DPI (p < 0.0001). Intestinal permeability at 8 DPI was significantly increased by 1.9-fold in infected mice compared to control group (p < 0.05) (Figure 1C). Hyperpermeability subsisted until at least 22 DPI (p < 0.05) and may involve a claudin-2 upregulation since mRNA level of this tight junction protein was increased in infected mice at 24 DPI, whereas occludin expression was significantly lowered (Figures 1D-E). No changes were observed for *ZO-1* mRNA level (Figures 1F). A semi-quantitative analysis of colonic damages has been performed (Table 1) on Hematoxylin, Phloxin, Safran (HPS) stained colon from control and infected group at 8 DPI and 24 DPI. We found that histological score increases significantly in infected mice compared to control group at 8 DPI and 24 DPI, mainly due to the presence of a marked colonic hyperplasia (Figure 1G). Histological examinations showed a marked colonic hyperplasia at 8 DPI, in the infected group (Figure 1H, middle panel) compared to the non-infected group (Figure 1H, left panel). After clearance of the pathogen (24 DPI), proximal colonic mucosa still exhibited hyperplasia characterized by an increase of crypts length (Figure 1H, right panel). No strong infiltration of immune cells was observed at any time.

**Table 1.** Histological score of colon mice

Evaluation criteria	Score	Characteristic(s)
Infiltration of inflammatory cells	0	No inflammatory cells in the lamina propria
	1	Increased numbers of inflammatory cells, including neutrophils in the lamina propria
	2	Confluence of inflammatory cells extending into the submucosa
	3	Transmural extension of the inflammatory cell infiltrate
Goblet cells	0	Presence of goblet cells in sufficient number
	1	Loss of goblet cells number
	2	Absence of goblet cell
Oedema size	0	No oedema
	1	Presence of a little oedema
	2	Presence of a big oedema
Epithelial damage	0	Absence of mucosal damage
	1	Discrete focal lympho-epithelial lesions
	2	Mucosal erosion/ulceration
	3	Extensive mucosal damage and extension through deeper structures of the bowel wall
Hyperplasia	0	No hyperplasia
	1	Low hyperplasia
	2	Moderate hyperplasia
	3	Severe hyperplasia

(a) Level of *C. rodentium* colonization followed by counting colony forming units (CFU) in the stools of infected mice (n = 8 per group) at different time points. (b) Low-grade inflammation assessed by measuring fecal lipocalin-2 at the peak of infection (7 DPI) and after clearance of the pathogen (23–24 DPI) (n = 12 per group). (c) Intestinal permeability assessed by measuring 4 kDa FITC-Dextran concentration in serum at the peak of infection (8 DPI) and after clearance of the pathogen (22 DPI) (n = 5–7 per group). (d-f) Colonic expression of (d) *occludin*, (e) *claudin-2* and (f) *ZO-1* mRNA in control (n = 10) and infected mice (n = 7–9) was quantified by RT-qPCR at 24 DPI. (g) Histological score and (h) histological analysis of colon by HPS staining in uninfected mice (left panel), *C. rodentium*-infected mice at 8 DPI (middle panel) and 23–24 DPI (right panel). Arrays represents crypts length. Scale bar = 50 µm. White circles represent control mice while black circles represent infected mice. Data are presented as mean ± SEM and are representative of two independent experiments. *p < 0.05 **p < 0.01 ****p < 0.0001.

### Citrobacter rodentium*-infected mice exhibited post-infectious CHS and anxiety-like behavior*

**Figure 2.**
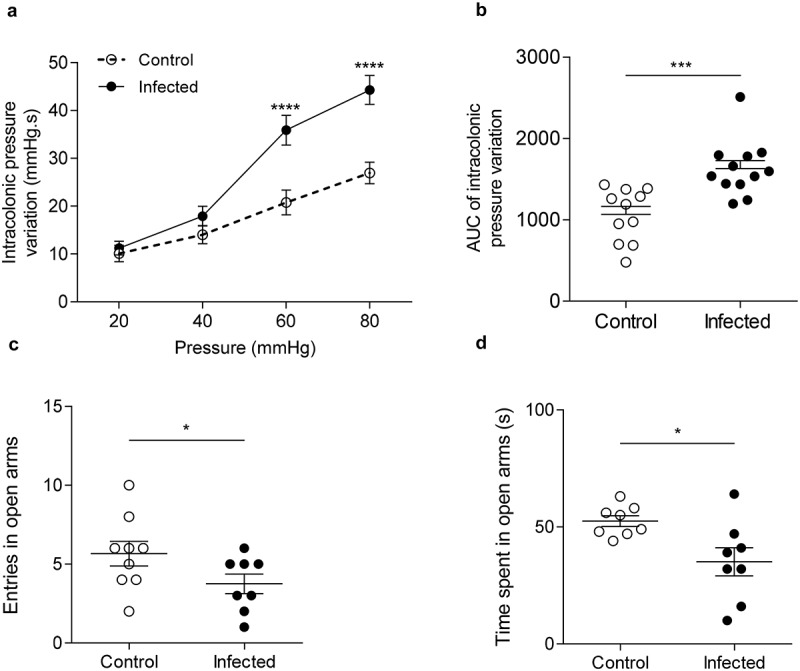
Post-infectious visceral sensitivity and anxiety-like behavior induced by *C. rodentium.*

At 24 DPI, intracolonic pressure variations, recorded as a surrogate marker of colonic sensitivity, from infected mice exhibited a 1.7-fold and 1.6-fold increase at 60 (p < 0.0001) and 80 mm Hg (p < 0.0001) distension pressures, respectively (Figure 2A **and S1A**). Corresponding areas under curve (AUC) were calculated for each mouse. The AUC average of infected group was significantly increased by 1.5-fold compared to AUC average of control group (p < 0.001), showing that *C. rodentium*-infected mice exhibited post-infectious CHS (Figure 2B). Infected mice also made significant fewer entries and spent less time in open arms of the EPM than control group (4.0 *versus* 6.0 entries, p < 0.05; 35.1 ± 6.4s *versus* 52.5 ± 2.3s, p < 0.05) (Figures 2C-D **and S1C-E**), revealing anxiety-like behavior.

(a) Post-infectious colonic sensitivity assessed by measuring intracolonic pressure variations in response to CRD and (b) corresponding AUC at 24 DPI (n = 11–12 per group). (C-D) Post-infectious anxiety-like behavior assessed with the elevated plus maze test. (c) Entries frequency and (d) time spent in open arms of control and infected mice at 21 DPI (n = 8–9 per group). White circles represent control mice and black circles represent infected mice. Data are presented as mean ± SEM and are representative of at least two independent experiments. *p < 0.05; ***p < 0.001; ****p < 0.0001.

### Citrobacter rodentium *infection induced long-term changes in composition and function of colonic mucosa-associated microbiota*

At 8 DPI, the number of observed operational taxonomic units was significantly lower in infected group (**Figure S2A**). Principal component analysis (PCoA) of beta-diversity clearly revealed that colonic mucosa-associated microbiota of infected mice was different from control mice (**Figure S2B**) (Adonis, p = 0.001). As expected, a significant increase of *Enterobacteriaceae* family relative abundance at the infection peak (p < 0.0001) was observed reflecting *C. rodentium* colonization (**Figure S2C**).^20^

**Figure 3.**
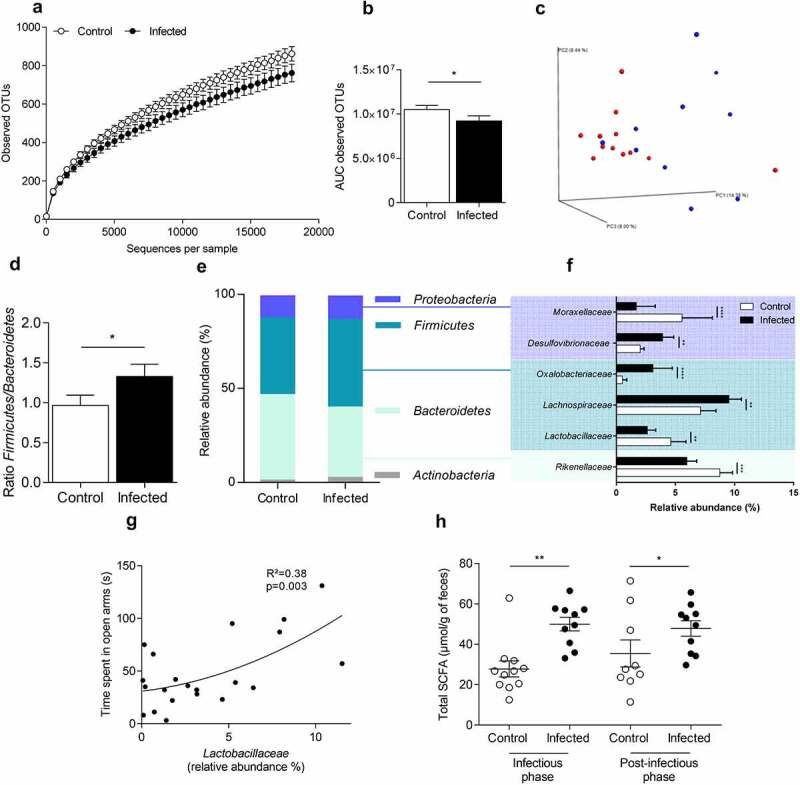
Colonic mucosa-associated microbiota dysbiosis after *C. rodentium* clearance in mice.

After clearance of the pathogen (24 DPI), bacterial richness was still decreased in comparison with control group (p < 0.05, Student’s t test) (Figure 3A-B), although PCoA of beta-diversity showed a weak population segregation between the two groups (Adonis, p = 0.055) (Figure 3C). A significant increase in *Firmicutes/Bacteroidetes* ratio was observed in infected mice (Figure 3D). Deeper analysis revealed four bacterial genera significantly modified in infected animals including a decrease in relative abundance of *Bacteroidetes* and increase in *Firmicutes, Proteobacteria*, and *Actinobacteria* (Figure 3E). At the family level, we observed a significant decrease in the relative abundance of *Moraxellaceae, Lactobacillaceae* and *Rikenellaceae* (p < 0.0001; p < 0.01 and p < 0.001, respectively) whereas *Desulfovibrionaceae, Oxalobacteracea* and *Lachnospiraceae* were increased (p < 0.01; p < 0.0001 and p < 0.01, respectively) (Figure 3F). Time spent in open arm was significantly positively correlated with the relative abundance of *Lactobacillaceae* (R^2^=0.38; p = 0.003) but not with *Moraxellaceae, Desulfovibrionaceae, Oxalobacteiaceae, Lachnospiraceae*, and *Rikenellaceae*) (Figure 3G).

After clearance of *C. rodentium* (24 DPI), the composition of the colonic mucosa-associated microbiota was determined using 16S DNA sequencing (V4-V5 regions) (n = 10–11 per group). (a) Alpha diversity was determined by observed Operational Taxonomic Units (OTUs) measurement according to sequences per sample of control and infected mice. (b) Area under curve (AUC) of the observed OTUs for control and infected mice were calculated. (c) Principal coordinates analysis (PCoA) of the unweight UniFrac distance of control (red plots) and infected mice (blue plots). Significance was assessed with Adonis (p = 0,055). (d) Determination of Ratio *Firmicutes/Bacteroidetes* in control and infected mice. (e-f) Relative abundance (as a percentage) of phyla (e) and family (f) which are significantly modified in infected mice at 24 DPI. (g) Correlation between time spent in open arms (elevated plus maze test) and relative abundance of *Lactobacillaceae* (R^2^=0,3845; p = 0,003). (h) Fecal concentrations of SCFA were measured by gas chromatography for infected and control animals at the peak of infection (7 DPI) and after the clearance of *C. rodentium* (24 DPI) (n = 10–12 per group). White bars and circles represent control mice while black bars and circles represent infected mice. *p < 0.05; **p < 0.01; ***p < 0.001; ****p < 0.0001.

Fecal concentration of SCFAs was measured at 7 and 24 DPI (Figure 3H). At the peak of infection, total SCFA concentration was enhanced by 1.8-fold in feces from infected mice (p < 0.001), with a significant increase of acetic and propionic acid (p < 0.001) (**Figures S3A)**. After clearance of the pathogen, total SCFA concentration remained higher in feces of infected mice (increased by 1.3-fold p < 0.05) (Figure 3H). However, this difference is not due to changes in the main SCFAs (acetic or propionic acid and butyrate) (**Figure S3B**).

### Citrobacter rodentium *infection induced long-term alterations of tryptophan metabolism*

**Figure 4.**
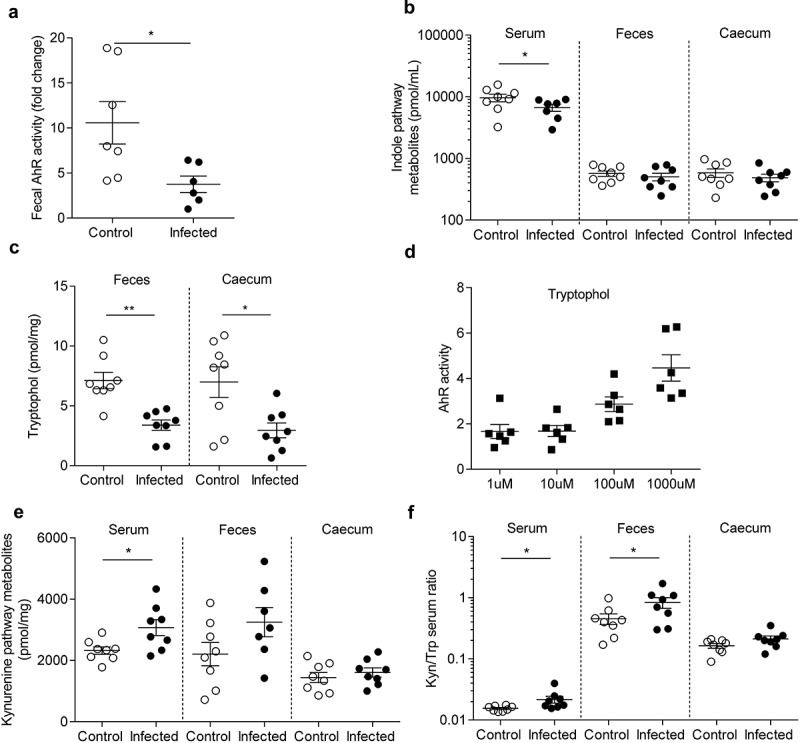
AhR activity and tryptophan metabolic pathways are modified in post-infectious period.

At 24 DPI, fecal AhR activity is significantly reduced in infected group (p < 0.05) (Figure 4A). Trp levels were unchanged between infected and control mice, whatever the studied matrix (serum, feces and caecal content) (**Figure S4A**). Indole pathway is decreased in serum from infected mice (p < 0.05) (Figure 4B). Deeper-specific analysis of each metabolite showed a significant decrease of tryptophol in feces (p < 0.01) and in caecum (p < 0.05) of infected mice (Figure 4C). AhR activity assay after a stimulation with increasing tryptophol doses showed that this indole metabolite activates AhR (Figure 4D). Kynurenine pathway was significantly increased in serum (p < 0.05) of infected mice but not in feces or cecum (Figure 4E). Even if Trp levels were unchanged, fecal and serum kynurenine/Trp ratio were significantly increased in infected mice (p < 0.05) (Figure 4F). No changes were observed for the serotonin pathway in any compartment (**Figure S4B**).

Tryptophan and its catabolites levels were measured by liquid chromatography coupled with high resolution mass spectrometry in three different compartments of control and infected mice after the clearance of the pathogen (24 DPI) (n = 8 per group). (a) Fecal AhR activity, which reveal the amount of AhR ligands, was assessed in feces using HepG2-Lucia™ AhR reporter cells. Results were normalized on the basis of negative luciferase activity of the control, cytotoxicity measurement and feces weight. (b) Indole pathway metabolites were quantified in the serum, feces and caecum. (c) Tryptophol was quantified in feces and caecum. (d) Dose-effect of tryptophol on AhR activity was assessed. (e) Kynurenine pathway metabolites were quantified in serum, feces and caecum. (f) Kynurenine/tryptophan ratio in serum, feces and caecum was evaluated for each group. White circles represent control mice while black circles represent infected mice. Data are presented as mean ± SEM and each plot represent a mouse. * p < 0.05; ** p < 0.01.

### *IL-22 treatment improved* Citrobacter rodentium*-induced anxiety-like behavior*

Colonic *IL-22* mRNA level was reduced during post-infectious period (24 DPI) (**Figure S5A**). IL-22 production was induced in intestinal epithelial cells by a daily oral gavage with *L. lactis* carrying IL-22 expression vector (*L. lactis*^IL-22^). This treatment was validated by an increase of *IL-22* mRNA level in colonocytes of mice treated with *L. lactis*^IL-22^ compared to mice gavaged with *L. lactis* carrying empty plasmid (*L. lactis*^empty^) (**Figure S5B**). None of the treatments affected body weight neither induced mortality nor toxicity in mice (data not shown). No effect of *L. lactis*^empty^ was detected in comparison with untreated mice on colonic sensitivity (**Figure S6A**) or on anxiety-like behavior (**Figure S6B**). Moreover, gastrointestinal tract colonization of mice infected with *C. rodentium* was similar for *L. lactis*^empty^ or *L. lactis*^IL-22^ treated mice (**Figure S6C**).

**Figure 5.**
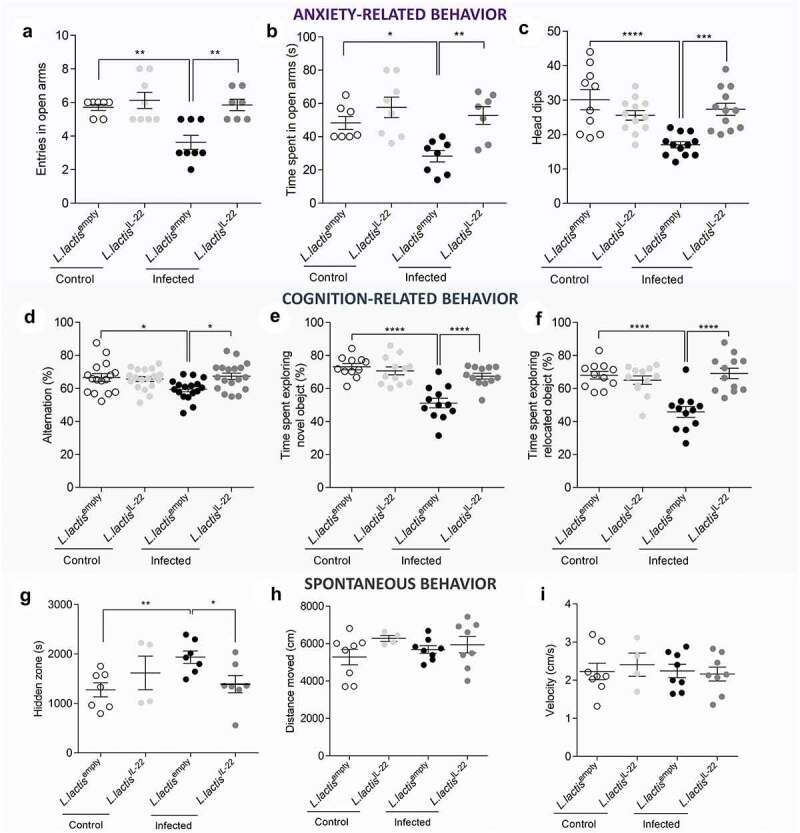
IL-22 treatment reverses post-infectious anxiety-, cognition-like behaviors and ill-being induced by *C. rodentium* infection.

Regarding anxiety-like behavior, the EPM test showed that infected mice treated with *L. lactis*^empty^ made significantly less entries in open arms compared to uninfected mice (p < 0.01) and also in comparison with infected mice treated with *L. lactis*^IL-22^ (p < 0.001) (Figure 5A **and S1D-F**). In addition, infected mice treated with *L. lactis*^IL-22^ spent an average of 51 ± 4.8s in open arms, which is comparable to control mice treated with *L. lactis*^empty^ (48 ± 4.0s). However, infected mice treated with *L. lactis*^empty^ exhibited a significant decrease of this duration (28.3 ± 3.5s) in comparison with control mice treated with *L. lactis*^empty^ (48.3 ± 4.0s) (p < 0.05) but also with infected mice treated with *L. lactis*^IL-22^ (51.3 ± 4.9s) (p < 0.01) (Figure 5B). Using the hole board test as another anxiety-related behavior assessment, infected mice treated with *L. lactis*^empty^ made significantly less head dips (17.0 ± 3.2) compared to uninfected mice treated with *L. lactis*^empty^ (30.1 ± 9.7) (p < 0.0001), or treated with *L. lactis*^IL-22^ (25.6 ± 4.9) (p < 0.01), and infected mice treated with *L. lactis*^IL-22^ (27.3 ± 6.1) (p < 0.001) (Figure 5C).

After a treatment with *L. lactis*^IL-22^, anxiety-related behavior induced by a *C. rodentium* post-infection were determined by (a-b) EPM test at D21 (n = 7–8 per group). (a) Entries frequency and (b) time spent in open arms (monitoring for 5 minutes). (c) Number of head dips made on the hole board for 5 minutes were quantified (n = 10–12 per group). Cognition-related behaviors analysis were performed using (d) Y-maze test in which spontaneous alternation were measured for 10 minutes and (e-f) time spent exploring the novel object and the relocated object during novel object and relocation tests, respectively. (g-i) Mice specific behaviors assessed using PhenoTyper® at 23 DPI and recorded for 24 hours (n = 4–8 mice per group). (g) Time spent inside the hidden zone, (h) distance moved and (**I**) velocity were determined during the activity period (12 hours dark period). White squares represent uninfected mice treated with *L. lactis*^empty^ and light grey squares represent uninfected mice treated with *L. lactis*^IL-22^ while black squares represent infected mice treated with *L. lactis*^empty^ and dark grey squares described infected mice treated with *L. lactis*^IL-22^. Data are representative of two independent experiments. *p < 0.05; **p < 0.01; ***p < 0.001 ****p < 0.0001.

No depression-like behavior wase developed in this post-infectious model as revealed by tail suspension (23 DPI) and forced swimming test (24 DPI) (**Figure S7A-B**). However, in post-infectious period, cognitive performances were affected in infected mice treated with *L. lactis*^empty^ in comparison with uninfected controls also treated with *L. lactis*^empty^. During the Y maze test, the percent of alternation was significantly decreased for infected mice treated with *L. lactis*^empty^ (59.4 ± 6.3%) in comparison with controls (66.5 ± 9.5%) (p < 0.05). This parameter returned to a normal spontaneous alternation in infected mice after a treatment with *L. lactis*^IL-22^ (67.3 ± 8.3%) (p < 0.05) (Figure 5D). This pattern of results was echoed by the novel object and location recognition tests in which infected mice treated with *L. lactis*^empty^ spent less time exploring both novel object (51.1 ± 2.9s *vs* 73.2 ± 1.9s p < 0.0001) (Figure 5E) and relocated object (45.8 ± 3.3s *vs* 68.1 ± 2.3s p < 0.0001) compared to uninfected control mice treated with *L. lactis*^empty^ (Figure 5F). IL-22 vectorization in infected mice significantly restored both exploration times (67.5 ± 1.8s exploring novel object and 69.1 ± 3.1s exploring relocated object p < 0.0001).

Spontaneous mouse behavior was analyzed at 23 DPI using a videotracking device system. Infected mice treated with *L. lactis*^empty^ spent more time in hidden zone during dark period in comparison with both uninfected mice (p < 0.01) and infected mice treated with *L. lactis*^IL-22^ (p < 0.05) (Figure 5G). Locomotion, distance, and velocity were not affected in any mice groups for the 24 hours analysis (Figure 5H-I).

### *IL-22 treatment improved* Citrobacter rodentium*-induced intestinal barrier disruption and CHS without change in intestinal inflammation*

**Figure 6.**
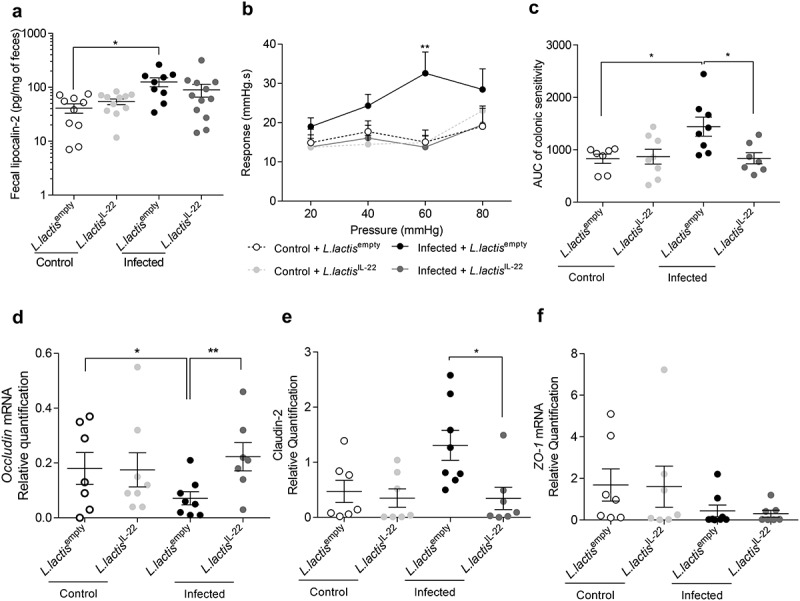
IL-22 treatment reverses post-infectious CHS and intestinal barrier alteration induced by *C. rodentium* infection independently of inflammation.

Low-grade inflammation was assessed using fecal lipocalin-2 measurement. An increase of this parameter was again observed in *C. rodentium* infected mice treated with *L. lactis*^empty^ compared to controls (125.6 ± 23.8 pg/mg of feces *vs* 41.1 ± 8.2 pg/mg of feces p < 0.05) (Figure 6A). However, *L. lactis*^IL-22^ treatment did not reverse this low-grade inflammation due to infection (89.5 ± 23.8 pg/mg of feces).

After a treatment with *L. lactis*^IL-22^, (a) low-grade inflammation assessed by measuring fecal lipocalin-2 (24 DPI) (n = 9–12 per group). (b-c) Post-infectious colonic sensitivity using CRD response at D24 (n = 7–8 per group). (b) Intracolonic pressure variations in response to CRD and (c) corresponding AUC. Colonic expression of (d) *occludin*, (e) *claudin-2* and (f) *ZO-1* mRNA was quantified by RT-qPCR at 24 DPI (n = 7–8 per group). White squares represent uninfected mice treated with *L. lactis*^empty^ and light grey squares represent uninfected mice treated with *L. lactis*^IL-22^ while black squares represent infected mice treated with *L. lactis*^empty^ and dark grey squares described infected mice treated with *L. lactis*^IL-22^. Data are representative of two independent experiments. *p < 0.05; **p < 0.01.

*Citrobacter rodentium*-infected mice treated with *L. lactis*^IL-22^ exhibited a 2.3-fold decrease response to 60 mm Hg distension pressures (p < 0.01) compared to infected mice treated with *L. lactis*^empty^ (Figures 6B). Infected mice treated with *L. lactis*^IL-22^ also showed a 1.7-fold decrease of colonic sensitivity AUC in comparison with infected mice treated with *L. lactis*^empty^ (Figures 6C **and S1B**).

The potential impact of this treatment on intestinal barrier integrity was explored by mRNA quantification of occludin, claudin-2 and ZO-1 tight junction proteins. *L. lactis*^IL-22^ treatment allowed infected mice to return to an *occludin* expression similar to uninfected mice (Figure 6D), as well as a decrease of *claudin-2* mRNA level (Figure 6E). No changes were observed for *ZO-1* mRNA level (Figure 6F).

## Discussion/Conclusion

One in ten patients develop IBS after an infectious illness, for which abdominal pain persists for several months or even years. Prospective studies show that 3% to 36% of enteric infections lead to persistent IBS symptoms. Whereas viral gastroenteritis seems to have only short-term effects, bacterial enteritis and protozoan or helminth infections are followed by prolonged PI-IBS. This infection can be caused by pathogenic *Enterobacteriaceae* as EHEC or EPEC.^4^ Disturbances of intestinal mucosa integrity and gut dysbiosis are possible involved mechanisms.^21^ Aims of this work were to clarify underlying mechanisms involved in PI-IBS using a mouse model of *C. rodentium* infection to mimic bacterial gastroenteritis in Humans. It may represent a relevant PI-IBS model as few studies demonstrated that infected mice exhibit CHS and gut dysbiosis at the peak of infection.^20,22–24^ Monitoring infection throughout our experiments using counting of CFU by plating fecal content on MacConkey agar allowed us to define the beginning of post-infectious period at 16 DPI, which is consistent with previous data observed with bioluminescence imaging of *C. rodentium* colonization.^25^ A study demonstrated long-lasting colonic hyperexcitability in *C. rodentium*-infected mice, 30 DPI, only after applying another acute stress.^26^ In this report, a persistent CHS (up to two weeks) was observed in infected mice after *C. rodentium* clearance, without adding acute stress. Some reports showed long-lasting colonic hyperexcitability *ex vivo*^23^ suggesting a persistent CHS while other reported transient CHS in *C. rodentium*-infected mice.^22^ As previously described,^27^ we observed that CHS was associated with stress-related disorders. Thus, anxiety-like behavior were assessed in C57BL6/J mice using the EPM reference test.^28^ After pathogen clearance, development of an anxiety-like behavior was observed in infected mice.^29^ In post-induced chronic colitis models, anxiety was observed as well as visceral hypersensitivity.^30,31^ However, other studies demonstrated that mice did not exhibit such behavior during infectious and post-infectious periods.^29,32^ Different mouse genetic backgrounds were used in those studies, which could affect behavior test outcome and thus explain these conflicting results. Cognitive impairments were exhibited in this *C. rodentium* mouse model^32^ as reflected by Y-maze and novel object/location recognition tests which. Spontaneous alternation was impaired by infection and restored with *L. lactis*^IL-22^ treatment. IL-22 vectorization also improved innate preference for novelty reflected by greater time spent exploring a novel object and a relocated object than a familiar one. Such treatment reduced cognition-related impairments, particularly recognition memory decrease, induced by a *C. rodentium* infection. No depression-related behavior was demonstrated in this post-infection model, which is not surprising since only 25% of IBS patients go to gastroenterology clinics with depression and 44% with anxiety.^33^ Chronic pain is clinically associated with the development of affective disorders.^34^ In a neuropathic pain model, affective consequences over time showed that mice developed anxiety-related behavior 4 weeks after induction of the neuropathy, and depression-related behaviors were observed after 6 to 8 weeks.^35^ These results suggest that anxiety can appear before depression.

Composition of the gut microbiota can influence cognitive functions as observed in several preclinical studies using GF mice and GI infection models.^3,32^ Recently, influence of tryptophan metabolism, particularly kynurenine pathway metabolites on brain function and behavior impairments, including anxiety, depression as well as cognitive performance and visceral pain perception has been the focus of increasing investigation.^9,12,27,36–41^ Analysis of associated-mucosa microbiota showed that *C. rodentium* infection induces colonic dysbiosis as observed in PI-IBS patients. Although ecosystem resilience may occur following recovery from an intestinal infection, patients developing PI-IBS may have an inability to restore microbial ecosystem due to host factors.^4^ In this study, a decrease of bacterial richness was observed in infected mice after clearance of the pathogen and their microbiota was different compared to uninfected mice microbiota. A decrease in the relative abundance of *Bacteroidetes* was observed as in IBS patients,^42^ which is supported by a significant increase in *Firmicutes/Bacteroidetes* ratio, a rough indicator of bacterial population shifts described in IBS patients.^43^ Abundance of *Firmicutes*, and more precisely of *Lachnospiraceae*, was increased in patients^44^ and same results were found in our post-infectious mice model. Abundance of the *Ruminococcaceae* family is also increased in post-infected mice. *Lachnospiraceae* and *Ruminococcaceae* families are both SCFAs-producers and we showed that fecal SCFAs production was increased in infected mice even after pathogen clearance, confirming previous observations in preclinical and clinical studies.^43,45^ Moreover, infected animals presented a decrease of *Lactobacillaceae* as reported in several analysis of gut microbiota in IBS patients.^43^ In order to better understand the mechanisms that lead to this dysbiosis, the use of gnotobiotic mouse model in which a minimal bacterial community is established in germ-free mice, such as Oligo-Mouse-Microbiota (Oligo-MM12)^46^ could be envisaged. In conclusion, even if the post-infectious duration is short, *C. rodentium* infection mouse model is currently a relevant animal model of PI-IBS, which is a complex and incompletely known pathology. Indeed, very few clinical studies distinguish IBS patients or PI-IBS patients, showing importance of this preclinical model to better understand the physiopathology of PI-IBS.

Our results showed that infection alters intestinal integrity, microbiota composition, function and notably microbial catabolism of tryptophan into AhR ligands, which could reduce the amount of colonic IL-22 even after pathogen clearance. In PI-IBS patients, increased intestinal permeability and low-grade inflammation were described.^2^ In the same way, we observed an intestinal permeability increase during infection, which persists after pathogen clearance. In our preclinical model, a significant increase of fecal lipocalin-2 expression was observed during infectious period, but also after *C. rodentium* clearance. Neutrophils are a source of lipocalin-2 and it has been proposed to use fecal lipocalin-2 as a biological marker for low-grade colonic inflammation.^47^ Moreover, neutrophils recruitment was previously described in *C. rodentium* infection model,^48^ and their activity has been shown to be increased and associated with neuronal excitability.^23^ In addition, a significant increase of the proinflammatory bacterial families, such as *Desulfovibrionaceae* was observed in post-infected mice in this study.^49^ These data suggest that *C. rodentium* infection causes development of low-grade inflammation, which could activate colonic nociceptors and thus trigger exaggerated nociceptive information to the central nervous system.^50^

Gut microbiota is considered as a key player in IBS physiopathology and is often associated with development of anxiety-like behaviors.^4^ Trp-derived metabolites are part of functional components in the microbiota-gut-brain axis. In feces and sera of post infected mice, the ratio kynureine/Trp was significantly increased. In the same way, clinical studies have shown that IBS patients have increased kynurenine concentrations compared to controls.^51,52^ Furthermore, a positive correlation was found between IBS severity (including CHS and anxiety) and the kynurenine/Trp ratio.^51^ This ratio reflects the indoleamine-2.3-dioxygenase (IDO) enzyme activity, which catalyzes the breakdown of Trp into kynurenine and whose expression is induced during inflammatory process. Comparing Trp metabolites, a decrease of indole metabolite level (indole-3-lactic acid) was observed in serum from women with IBS in comparison with healthy controls,^53^ which supported our significant decrease of indole metabolites in post-infected mice serum. Indeed, our results showed a decrease of indole pathway, with a specific reduction of tryptophol in feces and caecal content. Tryptophol activates AhR, which suggests a critical role of this indole metabolite in the pathophysiology of *C. rodentium* infection model and PI IBS. In our study, the post-infection dysbiosis is associated with a decrease of the *Lactobacillaceae* family. Interestingly, anxiety-like behavior was significantly anti-correlated with this *Lactobacillaceae* family decrease in *post-*infected mice. This lactic acid bacteria family uses tryptophan as a source of energy and produces AhR ligands that activate ILC3 and IL-22 release, which stimulates antimicrobial peptides production.^54^ Decreases of AhR ligands and IL-22 expression were observed after pathogen clearance. AhR signaling is considered as a key component of the immune response for gut barrier and is thus crucial for intestinal homeostasis by acting on epithelial renewal, barrier integrity, and many immune cell types, as intraepithelial lymphocytes, Th17 cells and innate lymphoid cells.^55^ AhR ligands produced by luminal gut microbiota can activate epithelial and immune cells in the gut wall^55^ but they can also reach nearby the enteric neurons and their projections to regulate the motor output of intestinal neural circuits.^11^ Our results support a role of Trp and its metabolites in PI-IBS physiopathology. We assumed that AhR/IL-22 pathway should have a critical role and thus targeting the IL-22 production could restore intestinal homeostasis, finally restoring the brain-gut axis communication.

In this study, a genetically modified lactic acid bacteria was used to locally deliver IL-22 cDNA expression vector directly in the intestine. This method allows an IL-22 low availability in intestinal epithelial cells. *Lactococcus lactis* is used as a lactic acid bacteria model because (i) its genome has been completely sequenced, (ii) it is easy to manipulate genetically, and (iii) several cloning vectors have been already developed.^4^ A study targeting the plasmid transfer efficiency using this *L. lactis* strain clearly showed that it is directly linked to the plasmid copy number level.^56,57^ Using this approach, we showed a local protective effect of IL-22 on mucosal epithelial barrier. *Citrobacter rodentium* infection disrupts intestinal permeability.^58^ The *L. lactis*^IL-22^ treatment restored colonic *occludin* mRNA level, which was decreased by infection, confirming the critical role of IL22 in maintaining intestinal barrier function.^59^ Such treatment improved CHS and anxiety-like behavior after pathogen clearance independently of infection-induced inflammation. Effects of infection and treatments were assessed with an original video-tracking device allowing a continuous and long-term monitoring of animal behavior, in a home cage-like setting. *C. rodentium* post-infected mice spent more time in the shelter zone during the dark period, which is positively associated with fear-related behavior and then be considered as anxiety- and depression-like troubles.^60^ Thus, the *L. lactis*^IL-22^ treatment favors healing of the main features caused by a *C. rodentium* infection in mice. These data confirmed the strong impact of AhR/IL-22 pathway deregulation in physiopathology of PI-IBS.

To summarize, the mouse model of *C. rodentium* infection is a relevant and predictive model to characterize underlying mechanisms of PI-IBS. This study highlights involvement of gut microbiota, since composition disturbances persist after pathogen clearance and correlations were found with cognitive impairments and anxiety-like behaviors. Moreover, Trp metabolism is altered during post-infectious period, especially through kynurenine and microbiota-associated indole pathways. In terms of therapeutic approaches, we have identified AhR/IL-22 signaling pathway as an important pharmacological target to reverse PI-IBS associated symptoms. Treatment approach resulting from our study is the possibility to use such *L. lactis*^IL-22^ treatment or to develop a therapeutic approach based on mRNA as developed for the SARS-CoV-2 vaccine^61^ to allow IL-22 production in intestinal epithelial cells of PI-IBS patients.

## Materials and methods

### Animals and ethic statement

Experimental protocols were approved by a local ethic committee (protocol number: EU0116-3460) and follow the guidelines of the Committee for Research and Ethical Issues of the International Association for the Study of Pain.^62^ Five-week-old specific pathogen-free C57BL6/J male mice were purchased from Janvier Labs (Le Genest-Saint-Isle, France). Mice were housed in animal biosafety level 2 (21–22°C, 12:12 h light-dark cycle), in the Animal Biosafety Level 2 (ABSL2) facility of University of Clermont Auvergne (Clermont-Ferrand, France), with access to food and water *ad libitum*.

### Citrobacter rodentium *infection*

*Citrobacter rodentium* strain (ATCC® 51459^TM^ DBS100) was grown overnight at 37°C in Luria Broth (Dutsher, Issy-les-Moulineaux, France) without shaking. Mice were then orally infected with 10^9^ CFU. Control mice were inoculated with 200 µl of sterile PBS. After infection, fecal samples were plated on MacConkey agar (Dutsher, Issy-les-Moulineaux, France) to count the CFU normalized to feces weight.

### IL-22 treatment

*L. lactis*^empty^ carries probiH1 plasmid. ProbiH1 is an *E. coli-L. lactis* shuttle plasmid containing RepA/RepC origin of replication, chloramphenicol resistance, and the eukaryotic expression cassette from pcDNA3 (Invitrogen). This eukaryotic expression cassette is composed of the Cytomegalovirus promoter (pCMV) a multiple cloning site and Bovine Growth Hormone polyadenylation site. Murine IL-22 sequence was cloned into ProbiH1 plasmid expression cassette, resulting in probiH1-IL-22, allowing thus the expression of IL-22 under the control of pCMV when the plasmid is inside eukaryotic cells and not prokaryotes like bacteria. ProbiH1-IL-22 was transformed in *L. lactis* MG1363. These lactic bacteria were used as a vector for DNA transfer. Mice were daily inoculated with 1 × 10^9^ CFU of either strain 16 days after *C. rodentium* infection and during 8 days. Control group was inoculated with *L. lactis*^empty^ while treated group was inoculated with *L. lactis*^IL-22^. Treatment began at the post-infectious phase, which is at 16 DPI.

### Colonic low-grade inflammation

Colonic low-grade inflammation was assessed by measuring fecal lipocalin-2 by ELISA.^47^ Fecal samples were collected at the peak of infection (7 DPI) and after clearance of the pathogen (23–24 DPI). Vials were centrifuged (10,000 x g, 10 minutes) and supernatants were stored at −20°C until analysis. Supernatants were then diluted in kit-recommended reagent (1% bovine serum albumin in PBS 1X) and concentration of lipocalin-2 was estimated using the Mouse Lipocalin-2/NGAL Duoset ELISA kit (R&D Systems, Minneapolis, MN) according to the manufacturer’s instructions. Plates were read at 450 nm (BioTekTM EpochTM, Thermo Fisher Scientific, Villebon sur Yvette, France). Concentrations were normalized to weight of feces.

### In vivo *intestinal permeability*

Four kDa FITC-dextran (TdB Consultancy AB, Uppsala, Sweden) was used as a marker of paracellular intestinal permeability. Mice were inoculated with 200 µl of FITC-Dextran (75 mg/ml in sterile 1X PBS) and replace in their own cage for 5 h. Mice were anesthetized with isoflurane (3% in O2) and 100 µl of blood were collected. Thirty minutes after samples were centrifuged (5000 x g, 30 minutes, 4°C) and FITC fluorescence (excitation 485 nm; emission 522 nm) in 50 µl of serum was measured using a microplate reader (Fluoroskan, Thermo Fisher Scientific, Villebon sur Yvette, France). Intestinal permeability was evaluated in terms of amount of FITC-dextran that had crossed the intestinal epithelial barrier to the blood after oral ingestion.

### Reverse transcription and quantitative polymerase chain reaction (RT-qPCR)

Total RNAs of proximal colon were extracted with Trizol (ThermoFisher Scientific, Waltham, MS, USA; Cat. No. 15,596,026). DNAse-treated RNAs were reverse-transcribed with high capacity cDNA RT kit (ThermoFisher Scientific, Waltham, MS, USA; Cat. No. 4,368,814) for RT-qPCR. Specific cDNA were amplified for occludin (Forward 5ʹ-AGTACATGGCTGCTGCTGATG-3ʹ; Reverse 5ʹ-CCCACCATCCTCTTGATGTGT-3), claudin-2 (Forward 5ʹ-ATGCCTTCTTGAGCCTGCTT-3ʹ; Reverse 5ʹ-AAGGCCTAGGATGTAGCCCA-3ʹ), ZO-1 (Forward 5ʹ-GTTCCGGGGAAGTTACGTGC −3ʹ; Reverse 5ʹ-AAGTGGGACAAAAGTCCGGG-3), IL-22 (Forward 5ʹ-TGTGCGATCTCTGATGGCTG-3ʹ; Reverse 5ʹ-GCTGGAAGTTGGACACCTCA-3ʹ) and 26S (Forward 5ʹ-TGTCATTCGGAACATTGTAG-3ʹ; Reverse 5ʹ-GGCTTTGGTGGAGGTC-3ʹ). qPCR assays were performed with SsoAdvanced Universal SYBR Green Supermix (Biorad, Hercules, CA, USA; Cat. No. 1,725,271) and carried out on CFX96 Touch Real-Time PCR Detection System (Biorad, Hercules, CA, USA). Relative quantification of *occludin, claudin-2, ZO-1* and *IL-22* mRNA levels was expressed as fold-change, using the 2^−ΔΔCt^ method with *26S* as reference gene.

### Mucosa-associated microbiota analyses

DNA was extracted from distal colonic tissues collected at the peak of infection (8 DPI) and after clearance of *C. rodentium* (23–24 DPI) using the NucleoSpin® Tissue XS kit (Macherey-Nagel, Hoerdt, France) according to the manufacturer’s instructions. Illumina high throughput sequencing were performed by MRDNA lab (Shallowater, TX, USA) on a MiSeq® following the manufacturer’s guidelines. Briefly, the V4-V5 regions of the bacterial 16S rRNA gene was amplified using 515 F/806 R primer pair and HotStarTaq Plus Master Mix® Kit (Qiagen, Germantown, MD, USA) under the following conditions: denaturation 94°C/3 min, followed by 28 cycles of 94°C/30 sec, 53°C/40 sec, and 72°C/1 min, with a final elongation step 72°C/5 min. After quality checks, pooled PCR products were purified using calibrated Ampure XP® beads. Illumina sequencing was performed, and DNA libraries build by following Illumina TruSeq DNA library® preparation protocol. We performed microbiota analyses on Quantitative Insights Into Microbial Ecology (QIIME, version 1.8.0) software package. In summary, sequences were demultiplexed to remove barcodes and primers. Chimeric sequences were removed using USEARCH61. Sequences were clustered using USEARCH61 with a 97% homology threshold. Taxonomic analyses were performed using the Greengenes reference database (version 13–8). Alpha diversity measures the richness of single microbial taxa within a sample. Observed operational taxonomic unit (OTU) measurements were determined with QIIME using an OTU table rarefied at various depths. AUC were calculated for each rarefaction curve. Beta diversity measures the variation in microbiota composition between individual samples. Unweighted UniFrac distances between samples were computed to measure beta diversity with the rarefied OTUs count table. Principal coordinates analysis (PCoA) was used to further assess and visualize beta diversity. Groups were compared for distinct clustering with Adonis.

### SCFA quantitation

Fecal SCFA concentrations were determined as previously described.^63^ Briefly, each fecal supernatant was analyzed as to SCFA on a gas-chromatography (Agilent Technologies 6850 Network GC System, Agilent Technologies, Santa Clara, SA, USA) equipped with a split-splitless injector, a flame-ionization detector and a capillary column (30 m; 0.25 mm; 0.25 µm) impregnated with nitroterephtalic acid modified polyethylene glycol (Agilent J&W DB-FFAP column, 122–3232E, Agilent Technologies, Santa Clara, SA, USA). Carrier gas (helium) flow rate was 0.7 ml/min and inlet, column and detector temperatures were 175, 100 and 240°C, respectively. A Volatile Free Acid Mix (Ref. CRM46975, Sigma-Aldrich, Saint-Quentin Fallavier, France) was used as an internal standard. Data were collected and peaks integrated using OpenLAB software (Agilent Technologies, Santa Clara, SA, USA). Concentrations were normalized to weight of feces.

### Tryptophan metabolites measurements

Trp and 20 Trp metabolites were quantified by liquid chromatography coupled with high resolution mass spectrometry from 3 different matrices: serum, feces and caecal content as previously described.^64^ Among them, 4 metabolites were quantified for serotonin pathway: melatonine, N-acetyl-serotonine, serotonine, 5-OH-tryptophane. For the kynurenine pathway, 7 metabolites were measured 3-OH-kynurenine, picolinic acid, xanthurenic acid, quinolinic acid, kynurenine, kynurenic acid, 3-OH-anthranilic acid. Nine metabolites of indole pathway were analyzed, such as tryptamine, tryptophol, 5-OH-indole acetic acid, indole-3-sulfate, indole, indole-3-lactic acid, indole-3-aldehyde, indole-3-acetic acid, indole-3-acetamide.

### Measurement of AhR activity

The AhR activity of animal stool samples was measured using HepG2-Lucia™ AhR reporter cells (InvivoGen, France). Cells were seeded into a 96-well plate and stimulated with animal stool samples or different amounts of tryptophol for 24 hours. Luciferase activity was measured using a luminometer and Quanti-Luc reagent (InvivoGen). The results were normalized on the basis of the negative luciferase activity of the control, cytotoxicity measurement (CytoTox 96 Non-radioactive Cytotoxicity Assay, Promega) and feces weight.

### Anxiety-related behavioral tests

#### Elevated plus maze

Anxiety-like behavior was first assessed using the EPM test (ViewPoint Behavior Technology, Lissieu, France) at 21 DPI. The apparatus consists of two opposite open arms (37 × 6 × 0.6 cm) and two closed arms (37 × 6 × 15 cm), joined by a common central platform (15 × 15 cm), and subjected to an equal illumination (30 lux). The maze is elevated 50 cm above the floor. Mice were acclimated to the room at least 45 minutes before test. Individual animal was placed in the central zone and allowed to explore the maze for 5 minutes. Mice were recorded with a camera and data were manually scored. The perform distance in the apparatus was recorded (Ethovison XT 15, Noldus). Anxiety was characterized through the number of entries in each arm (considered when the four taws are located within the arm) and time spent in open arms.

#### Hole board test

The hole board test is used to evaluate the rodents’ emotionality, anxiety state, and/or stress responses response to an unfamiliar environment.^65^ The test consists of a board (39.5 × 39.5 cm) with 16 equidistant holes, 3 cm in diameter, equally distributed throughout the platform and placed 70 cm above the ground, with an equal illumination (30 lux). At 24 DPI, mice were acclimated to the room at least 45 minutes before test. Mice (n = 10–12 per group) were individually placed on one corner of the board facing away from the experimenter. The number of head dips in the holes were quantified for 5 minutes.

### Cognitive-related behavioral tests

#### Y-maze

The Y-maze apparatus is a spatial recognition memory test consisting of three identical arms (45 × 13 cm, with 45 cm high walls) that were intersected at 120° around a central triangle (Laurent et al., 2017). Mice were placed at the end of one arm and allowed to move freely through the maze for 10 minutes. Entries into all arms were noted (four paws had to be inside the arm for a valid entry) and a spontaneous alternation was counted if an animal entered three different arms consecutively. The distance moved in the apparatus was recorded (Ethovison XT 15, Noldus). Percentage of spontaneous alternation was calculated according to following formula: [(number of alternations)/(total number of arm entries − 2)] × 100.^66^

#### Novel object recognition (NOR) and novel location recognition (NLR)

The day before testing mice were individually placed into the open field arena (45 × 45 × 40 cm) for 10 min of habituation. On the testing day, mice (n = 11–12 per group) were placed in the arena containing two identical objects placed on opposite symmetrical corners for an acquisition trial of 10 min. Animals were then removed to their home cage for 1 h and placed again in the arena where one of the familiar object previously presented was randomly replaced by a novel object (NOR). Percentage of time spent exploring the novel object was determined. Exploration was defined as the orientation of animal’s snout toward the object, sniffing or touching with snout, while running around the object, sitting or climbing on it was not considered as exploration.^67^ After 1-h inter-trial time, animals were retested while the novel object was relocated in the opposite corner to the familiar object (NLR). Location of novel object versus familiar object was counterbalanced. Percentage of time spent exploring the novel object location was measured to determine spatial recognition memory.

### Depressive-related behavioral tests

#### Tail suspension test

Mice (n = 16–18 per group) were suspended by their tail on a piece of string maintained by masking tape placed approximately 1 cm below the tip of the tail and attached to a suspended bar placed at 30 cm above the bench.^68^ Time in total immobility was measured during a 5 min session test.

#### Forced swimming test

Mice were individually placed in a 16-cm diameter and 25-cm depth beaker filled with 20 cm of warm water (22–25°C) as previously described.^69^ Total time in immobility over a 5 min session was determined as depression-related behavior. Mice (n = 16–18 in each group) were considered motionless when only a slight move to stay afloat was observed.

### Spontaneous mouse behavior analyses

The PhenoTyper**®** (Noldus Information Technology, Wageningen, The Netherlands) is an automated infrared (IR) video-tracking system measuring behavior of animal models. This device is composed of 8 plexiglass cages (45 cm × 45 cm), each containing an opaque plastic square shelter accessible by two entrances and delimited areas for feeding and drinking. Cages are surrounded by top unit provided with IR lighting and the IR camera for video recording during the light and dark cycles. Briefly, at D23 PI, mice were transferred into PhenoTyper**®** cages (1 per cage) and animal activities were recorded with Mediarecorder**®** (Noldus Information Technology, Wageningen, Netherlands) for 24 h, including 12-h dark period and 12-h light period. During this period, mice had *ad libitum* access to food, water and a shelter. Raw data were analyzed with Ethovision XT**®** software (Version 12, Noldus Information Technology, Wageningen, Netherlands) and various specific behaviors were compared between infected and control animals.

### Colorectal distension test

Colonic sensitivity was assessed using non-invasive manometric method recently developed and validated in mice, as described previously with minor changes.^70^ Briefly, mice were anesthetized with isoflurane (3% in O_2_) and the lubricated “balloon-pressure” sensor catheter was introduced into the rectum so that the distal end of the balloon was positioned 1 cm from the anus. Each balloon was connected to the barostat. The CRD protocol consisted of graded phasic distensions to constant pressures of 20, 40, 60, and 80 mm Hg. Each CRD pressure was repeated twice and the increase in the AUC of intracolonic pressure during CRD was average for each pressure.

### Histopathology assessment

To assess the degree of colitis and hyperplasia, Hematoxylin, Phloxin, Safran (HPS) staining was performed according to standard procedures. Briefly, mice were anesthetized with isoflurane (3% in O_2_), proximal sections of colons were fixed in 4% paraformaldehyde for 24 h at 4°C followed by dehydration in sucrose 30% for 48 h. Samples were then frozen in O.C.T. compound (Tissue Freezing Medium, Microm Microtech, Brignais, France). Sections of 10 µm were cut using a cryostat (Leica CM1950, Leica Biosystems, Nanterre, France) and stained. Histological analyses were executed by a pathologist (Dr. Catherine GODFRAIND) using a semi-quantitative colitis histological score as previously described^9^ and reported in Table 1.

### Statistical analysis

Statistical analysis were performed with GraphPad Prism 7 software (GraphPad, La Jolla, USA). Mann-Whitney and Student’s t-tests were used to compare two groups. Kruskal-Wallis test with a Dunn’s multiple post hoc comparison test was used for comparison between more than two groups. Two-Way ANOVA test with a Sidak multiple post hoc comparison test was for multiple factors influencing. Pearson test was performed to determined correlations between two independent variables.

## Supplementary Material

Supplemental Material

## Data Availability

All sequencing raw data have been deposited in European Nucleotide Archive (ENA) under accession number PRJEB46366.
