# Supplementary material for: AhR/IL-22 pathway as new target for the treatment of post-infectious irritable bowel syndrome symptoms
Source: Gut Microbes. 2022 Jan 28;14(1):2022997. doi: 10.1080/19490976.2021.2022997 (PMC8803069; doi:10.1080/19490976.2021.2022997)
Supplement: Supplemental Material [file KGMI_A_2022997_SM9733.zip › supplementary/Revised Figures.pptx]

## Slide 1
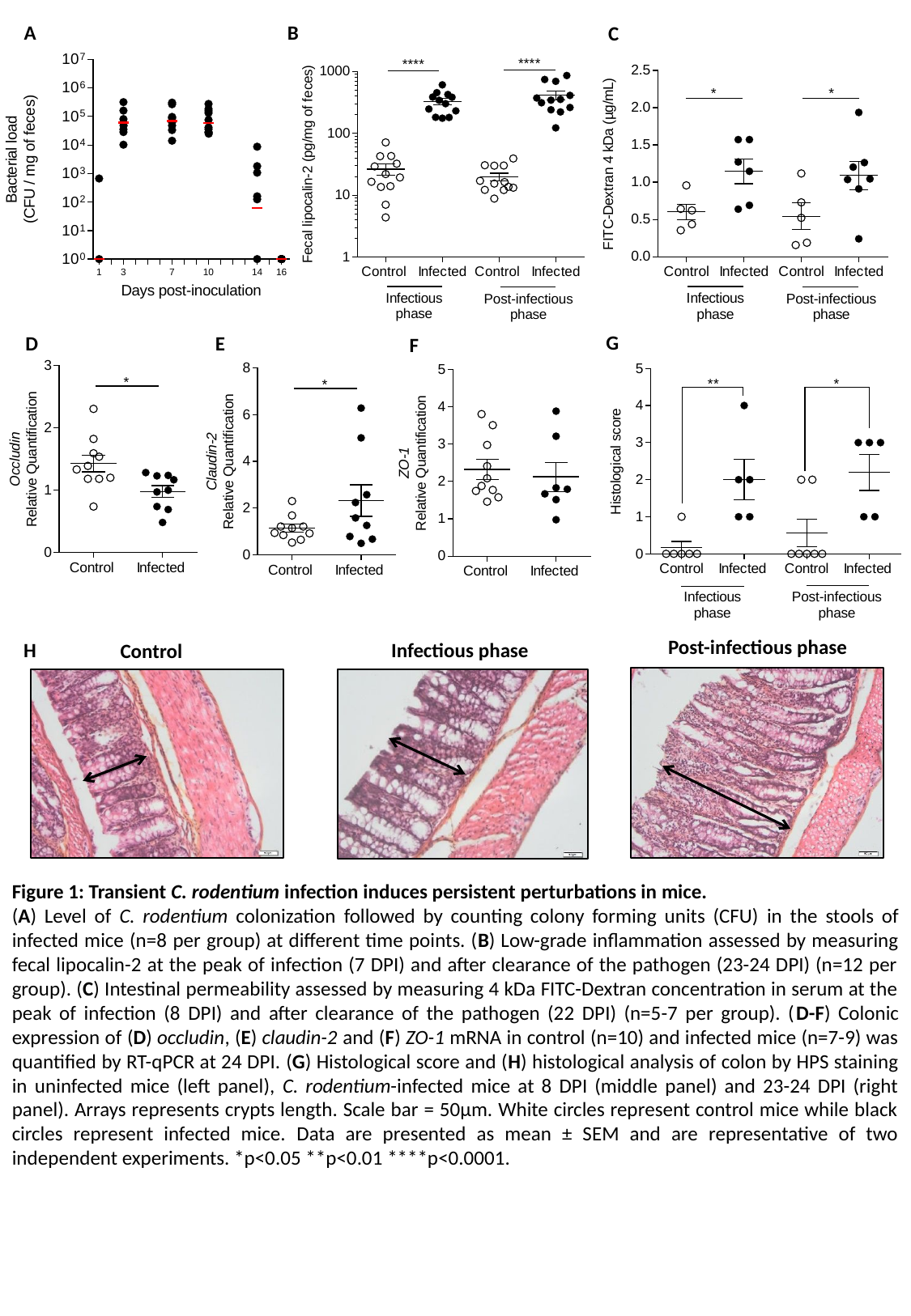

B
A
C
G
E
D
F
Post-infectious phase
Infectious phase
H
Control
Figure 1: Transient C. rodentium infection induces persistent perturbations in mice.
(A) Level of C. rodentium colonization followed by counting colony forming units (CFU) in the stools of infected mice (n=8 per group) at different time points. (B) Low-grade inflammation assessed by measuring fecal lipocalin-2 at the peak of infection (7 DPI) and after clearance of the pathogen (23-24 DPI) (n=12 per group). (C) Intestinal permeability assessed by measuring 4 kDa FITC-Dextran concentration in serum at the peak of infection (8 DPI) and after clearance of the pathogen (22 DPI) (n=5-7 per group). (D-F) Colonic expression of (D) occludin, (E) claudin-2 and (F) ZO-1 mRNA in control (n=10) and infected mice (n=7-9) was quantified by RT-qPCR at 24 DPI. (G) Histological score and (H) histological analysis of colon by HPS staining in uninfected mice (left panel), C. rodentium-infected mice at 8 DPI (middle panel) and 23-24 DPI (right panel). Arrays represents crypts length. Scale bar = 50µm. White circles represent control mice while black circles represent infected mice. Data are presented as mean ± SEM and are representative of two independent experiments. *p<0.05 **p<0.01 ****p<0.0001.

## Slide 2
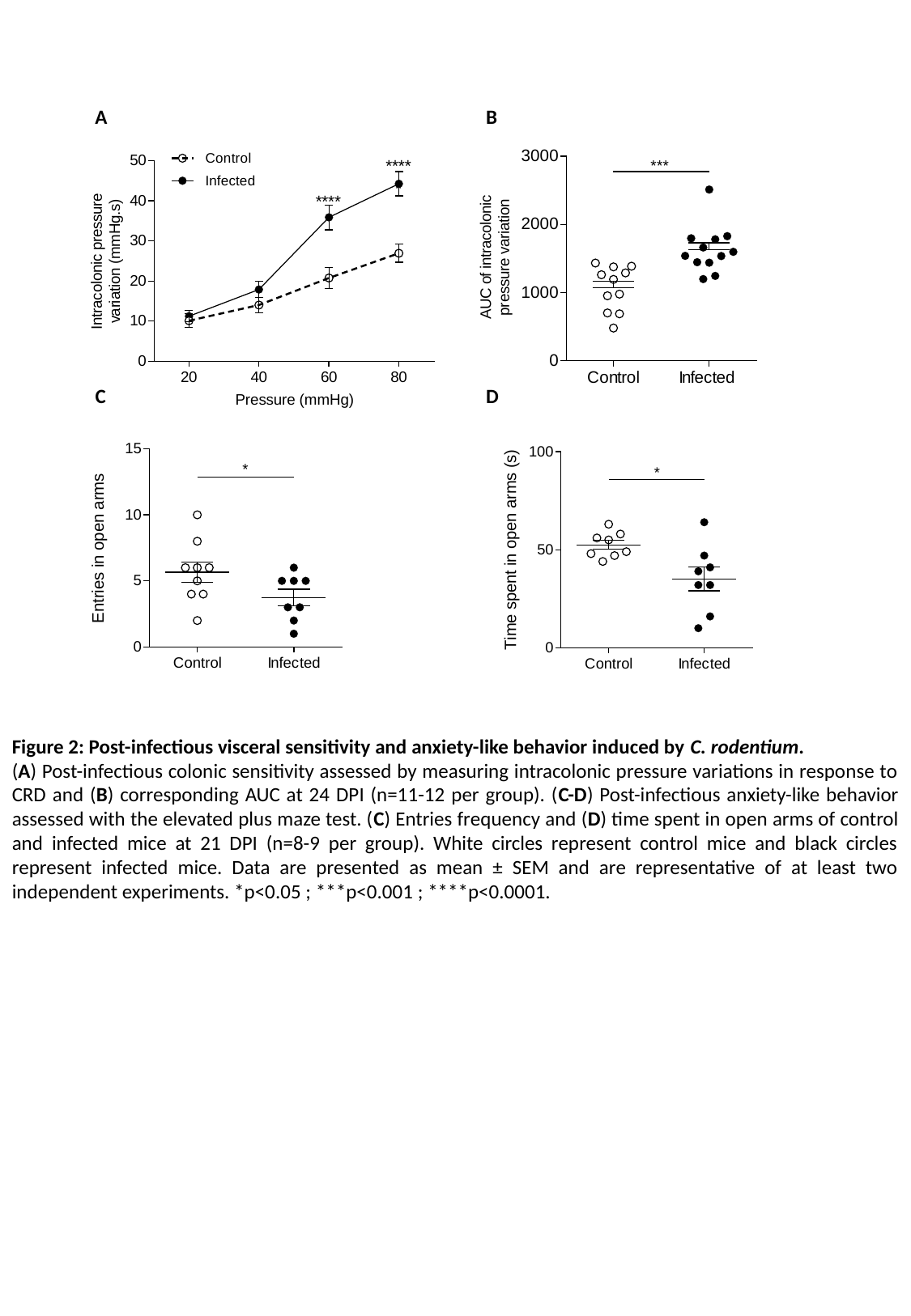

B
A
D
C
Figure 2: Post-infectious visceral sensitivity and anxiety-like behavior induced by C. rodentium.
(A) Post-infectious colonic sensitivity assessed by measuring intracolonic pressure variations in response to CRD and (B) corresponding AUC at 24 DPI (n=11-12 per group). (C-D) Post-infectious anxiety-like behavior assessed with the elevated plus maze test. (C) Entries frequency and (D) time spent in open arms of control and infected mice at 21 DPI (n=8-9 per group). White circles represent control mice and black circles represent infected mice. Data are presented as mean ± SEM and are representative of at least two independent experiments. *p<0.05 ; ***p<0.001 ; ****p<0.0001.

## Slide 3
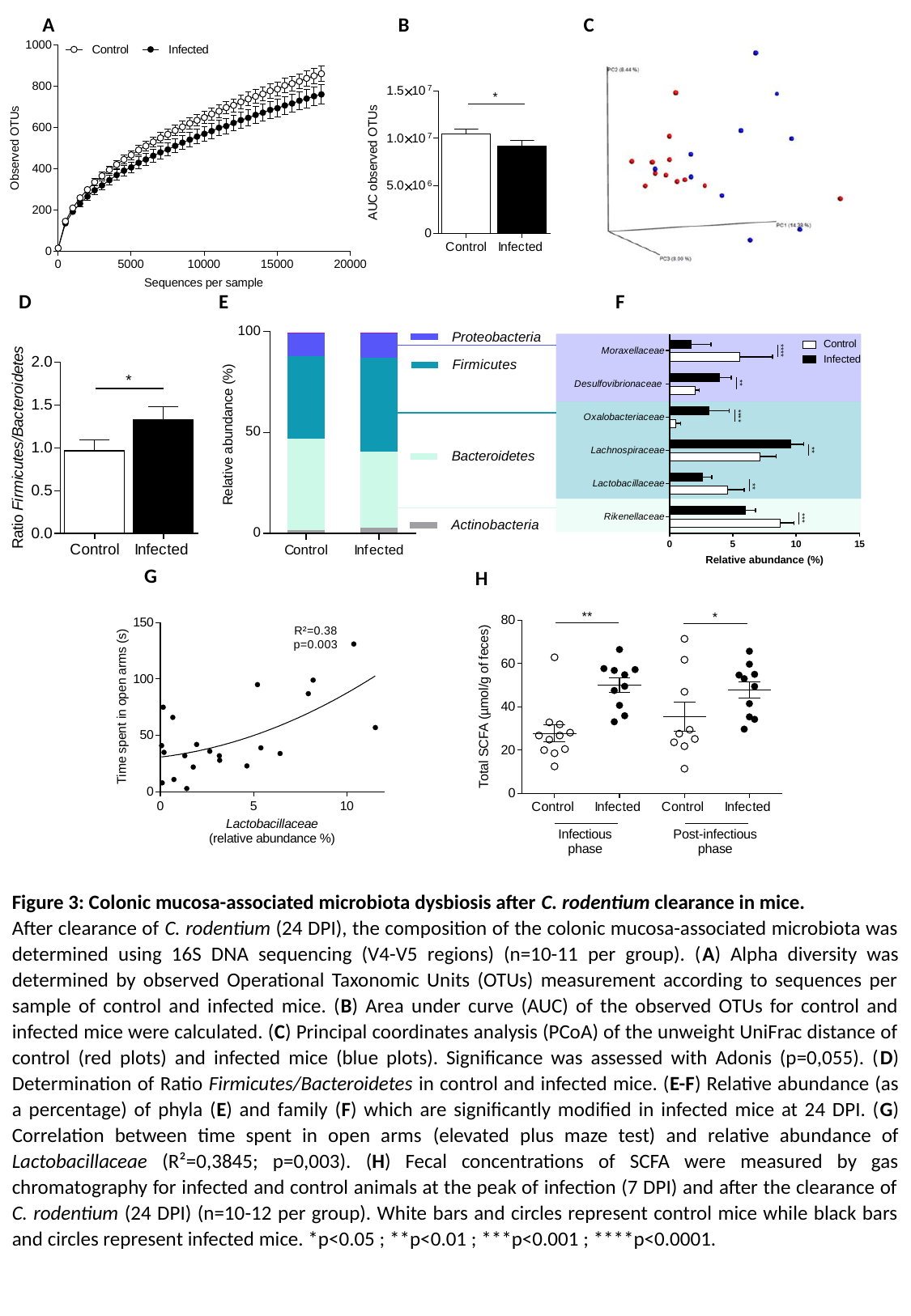

C
B
A
D
F
E
G
H
Figure 3: Colonic mucosa-associated microbiota dysbiosis after C. rodentium clearance in mice.
After clearance of C. rodentium (24 DPI), the composition of the colonic mucosa-associated microbiota was determined using 16S DNA sequencing (V4-V5 regions) (n=10-11 per group). (A) Alpha diversity was determined by observed Operational Taxonomic Units (OTUs) measurement according to sequences per sample of control and infected mice. (B) Area under curve (AUC) of the observed OTUs for control and infected mice were calculated. (C) Principal coordinates analysis (PCoA) of the unweight UniFrac distance of control (red plots) and infected mice (blue plots). Significance was assessed with Adonis (p=0,055). (D) Determination of Ratio Firmicutes/Bacteroidetes in control and infected mice. (E-F) Relative abundance (as a percentage) of phyla (E) and family (F) which are significantly modified in infected mice at 24 DPI. (G) Correlation between time spent in open arms (elevated plus maze test) and relative abundance of Lactobacillaceae (R²=0,3845; p=0,003). (H) Fecal concentrations of SCFA were measured by gas chromatography for infected and control animals at the peak of infection (7 DPI) and after the clearance of C. rodentium (24 DPI) (n=10-12 per group). White bars and circles represent control mice while black bars and circles represent infected mice. *p<0.05 ; **p<0.01 ; ***p<0.001 ; ****p<0.0001.

## Slide 4
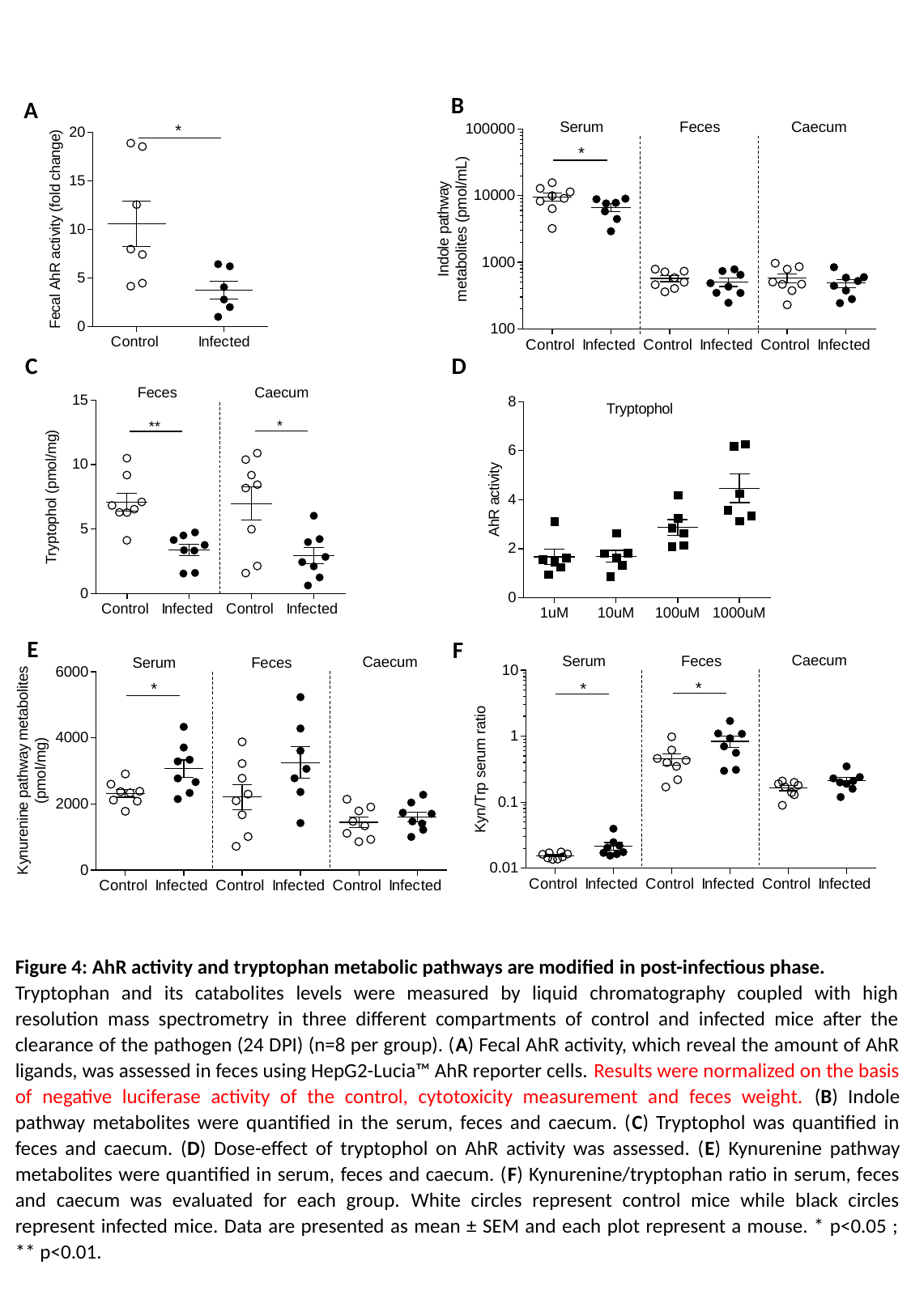

B
A
C
D
E
F
Figure 4: AhR activity and tryptophan metabolic pathways are modified in post-infectious phase.
Tryptophan and its catabolites levels were measured by liquid chromatography coupled with high resolution mass spectrometry in three different compartments of control and infected mice after the clearance of the pathogen (24 DPI) (n=8 per group). (A) Fecal AhR activity, which reveal the amount of AhR ligands, was assessed in feces using HepG2-Lucia™ AhR reporter cells. Results were normalized on the basis of negative luciferase activity of the control, cytotoxicity measurement and feces weight. (B) Indole pathway metabolites were quantified in the serum, feces and caecum. (C) Tryptophol was quantified in feces and caecum. (D) Dose-effect of tryptophol on AhR activity was assessed. (E) Kynurenine pathway metabolites were quantified in serum, feces and caecum. (F) Kynurenine/tryptophan ratio in serum, feces and caecum was evaluated for each group. White circles represent control mice while black circles represent infected mice. Data are presented as mean ± SEM and each plot represent a mouse. * p<0.05 ; ** p<0.01.

## Slide 5
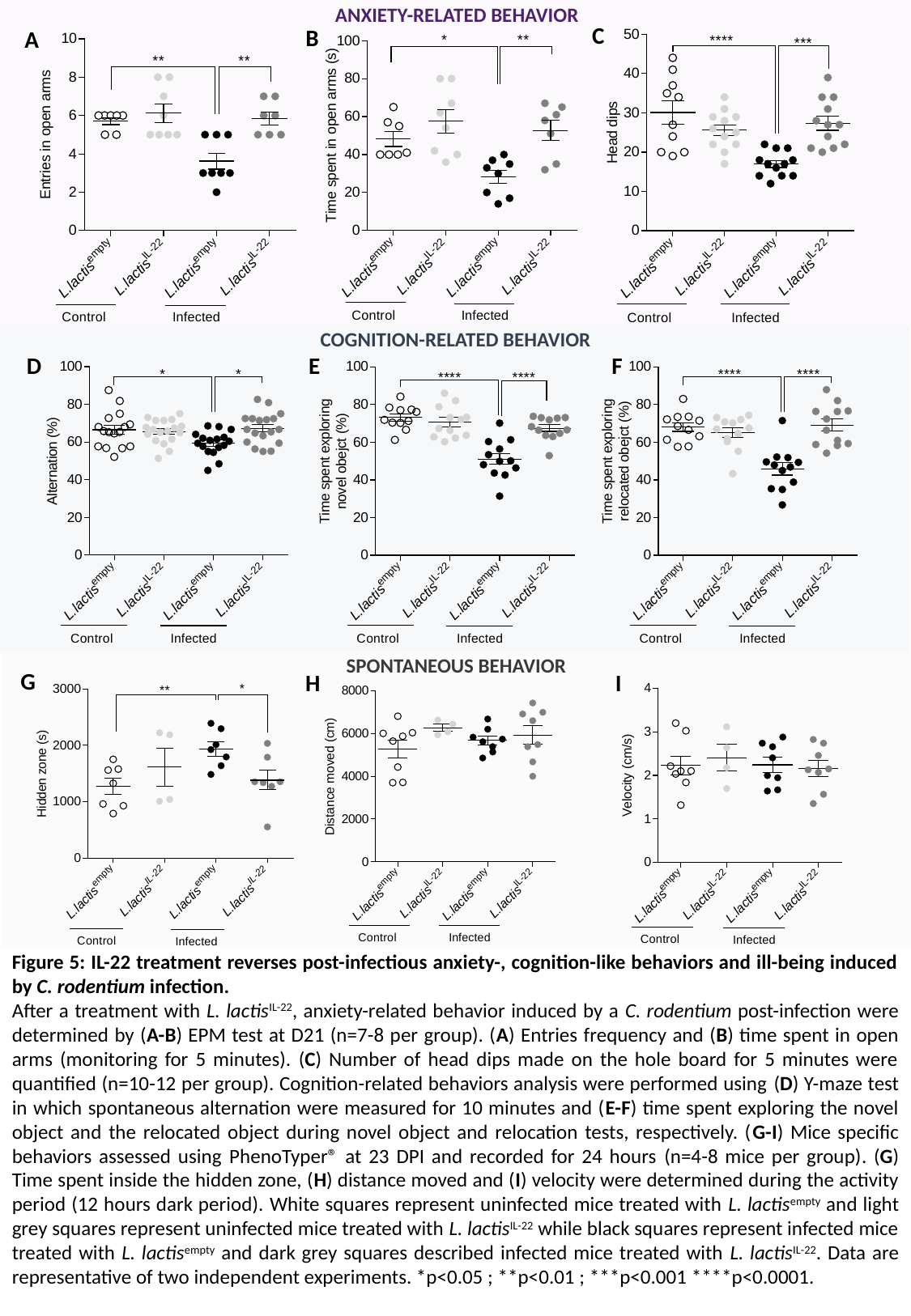

ANXIETY-RELATED BEHAVIOR
C
B
A
COGNITION-RELATED BEHAVIOR
D
E
F
SPONTANEOUS BEHAVIOR
G
H
I
Figure 5: IL-22 treatment reverses post-infectious anxiety-, cognition-like behaviors and ill-being induced by C. rodentium infection.
After a treatment with L. lactisIL-22, anxiety-related behavior induced by a C. rodentium post-infection were determined by (A-B) EPM test at D21 (n=7-8 per group). (A) Entries frequency and (B) time spent in open arms (monitoring for 5 minutes). (C) Number of head dips made on the hole board for 5 minutes were quantified (n=10-12 per group). Cognition-related behaviors analysis were performed using (D) Y-maze test in which spontaneous alternation were measured for 10 minutes and (E-F) time spent exploring the novel object and the relocated object during novel object and relocation tests, respectively. (G-I) Mice specific behaviors assessed using PhenoTyper® at 23 DPI and recorded for 24 hours (n=4-8 mice per group). (G) Time spent inside the hidden zone, (H) distance moved and (I) velocity were determined during the activity period (12 hours dark period). White squares represent uninfected mice treated with L. lactisempty and light grey squares represent uninfected mice treated with L. lactisIL-22 while black squares represent infected mice treated with L. lactisempty and dark grey squares described infected mice treated with L. lactisIL-22. Data are representative of two independent experiments. *p<0.05 ; **p<0.01 ; ***p<0.001 ****p<0.0001.

## Slide 6
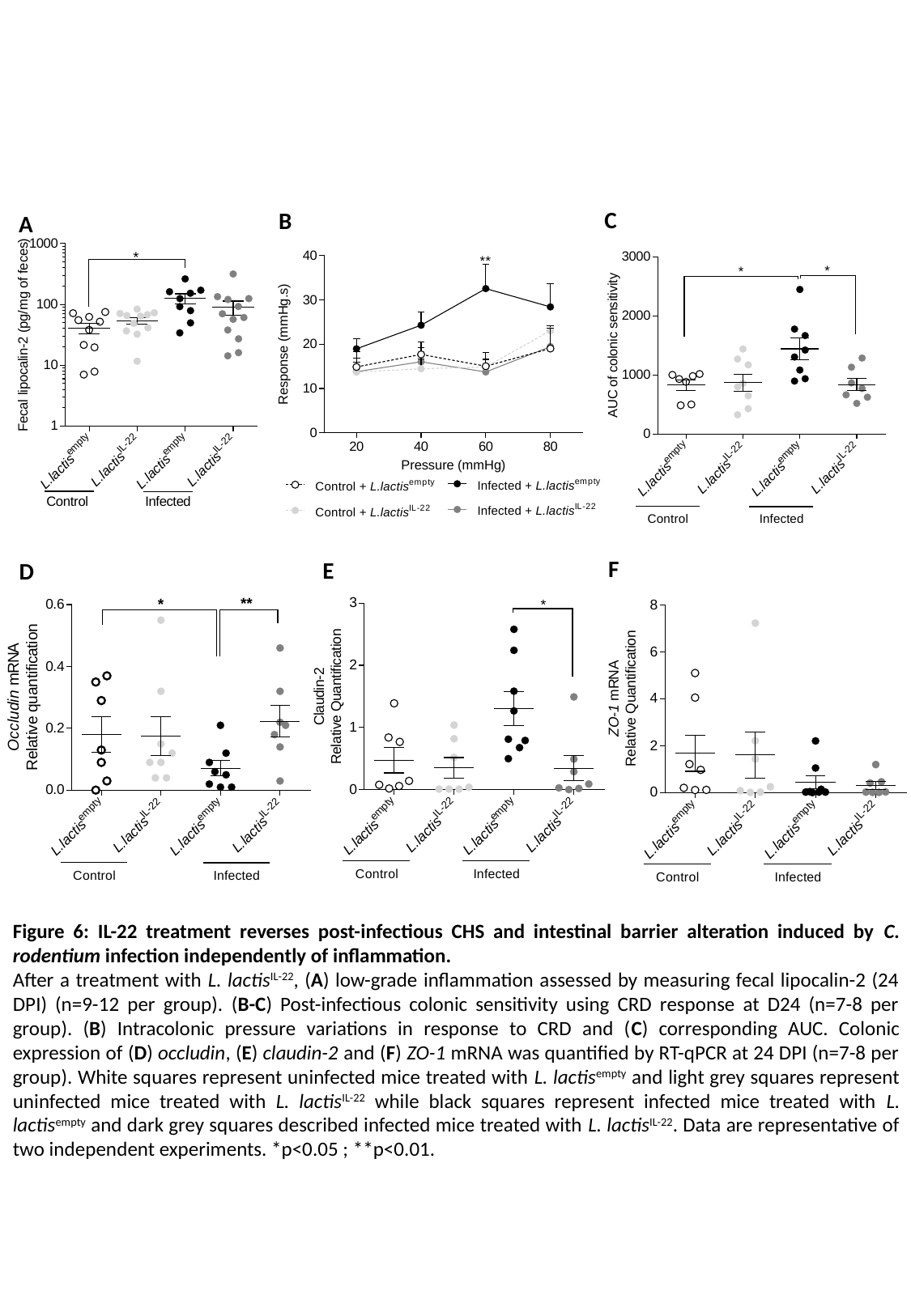

C
B
A
F
E
D
Figure 6: IL-22 treatment reverses post-infectious CHS and intestinal barrier alteration induced by C. rodentium infection independently of inflammation.
After a treatment with L. lactisIL-22, (A) low-grade inflammation assessed by measuring fecal lipocalin-2 (24 DPI) (n=9-12 per group). (B-C) Post-infectious colonic sensitivity using CRD response at D24 (n=7-8 per group). (B) Intracolonic pressure variations in response to CRD and (C) corresponding AUC. Colonic expression of (D) occludin, (E) claudin-2 and (F) ZO-1 mRNA was quantified by RT-qPCR at 24 DPI (n=7-8 per group). White squares represent uninfected mice treated with L. lactisempty and light grey squares represent uninfected mice treated with L. lactisIL-22 while black squares represent infected mice treated with L. lactisempty and dark grey squares described infected mice treated with L. lactisIL-22. Data are representative of two independent experiments. *p<0.05 ; **p<0.01.

## Slide 7
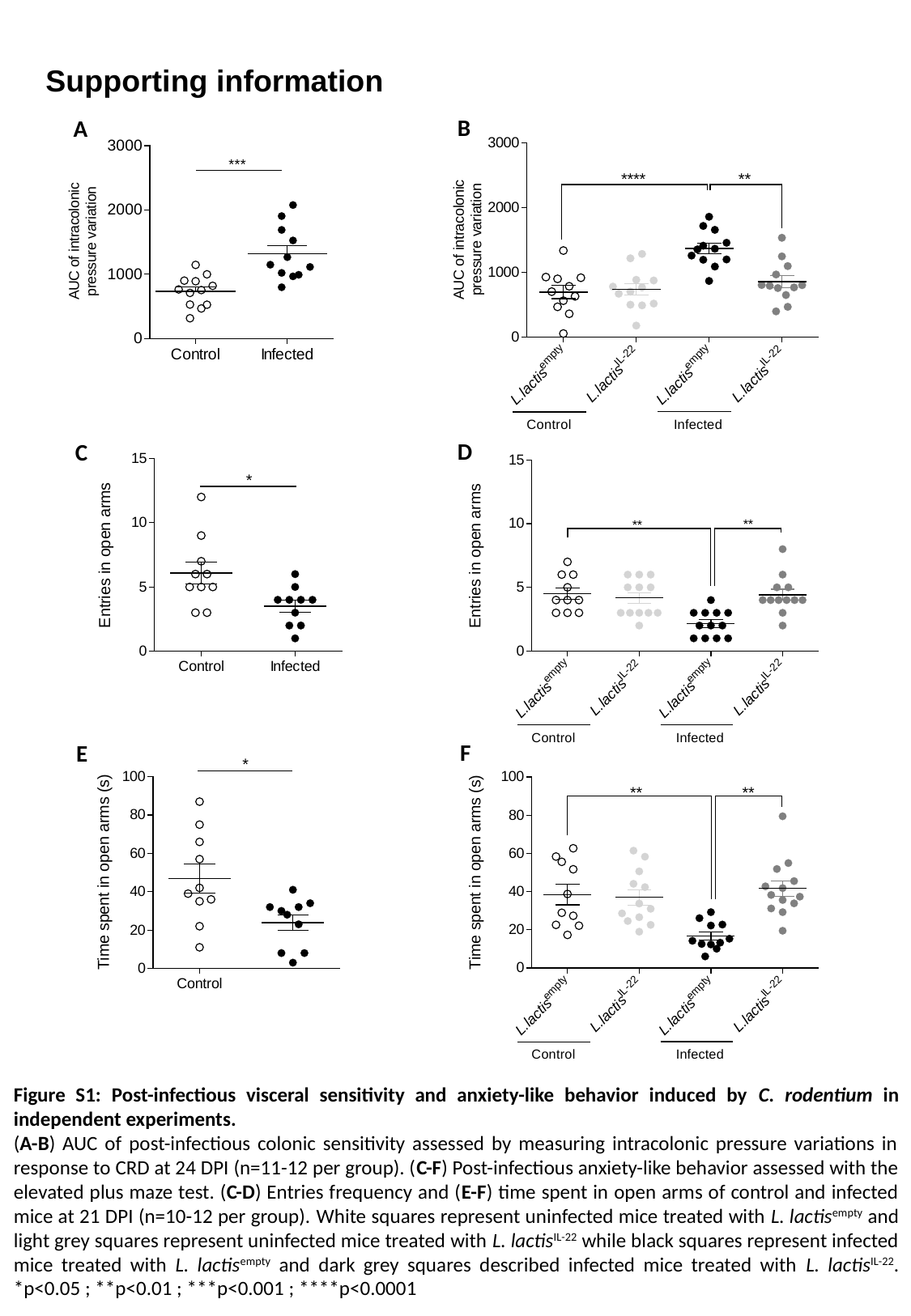

Supporting information
B
A
D
C
F
E
Figure S1: Post-infectious visceral sensitivity and anxiety-like behavior induced by C. rodentium in independent experiments.
(A-B) AUC of post-infectious colonic sensitivity assessed by measuring intracolonic pressure variations in response to CRD at 24 DPI (n=11-12 per group). (C-F) Post-infectious anxiety-like behavior assessed with the elevated plus maze test. (C-D) Entries frequency and (E-F) time spent in open arms of control and infected mice at 21 DPI (n=10-12 per group). White squares represent uninfected mice treated with L. lactisempty and light grey squares represent uninfected mice treated with L. lactisIL-22 while black squares represent infected mice treated with L. lactisempty and dark grey squares described infected mice treated with L. lactisIL-22. *p<0.05 ; **p<0.01 ; ***p<0.001 ; ****p<0.0001

## Slide 8
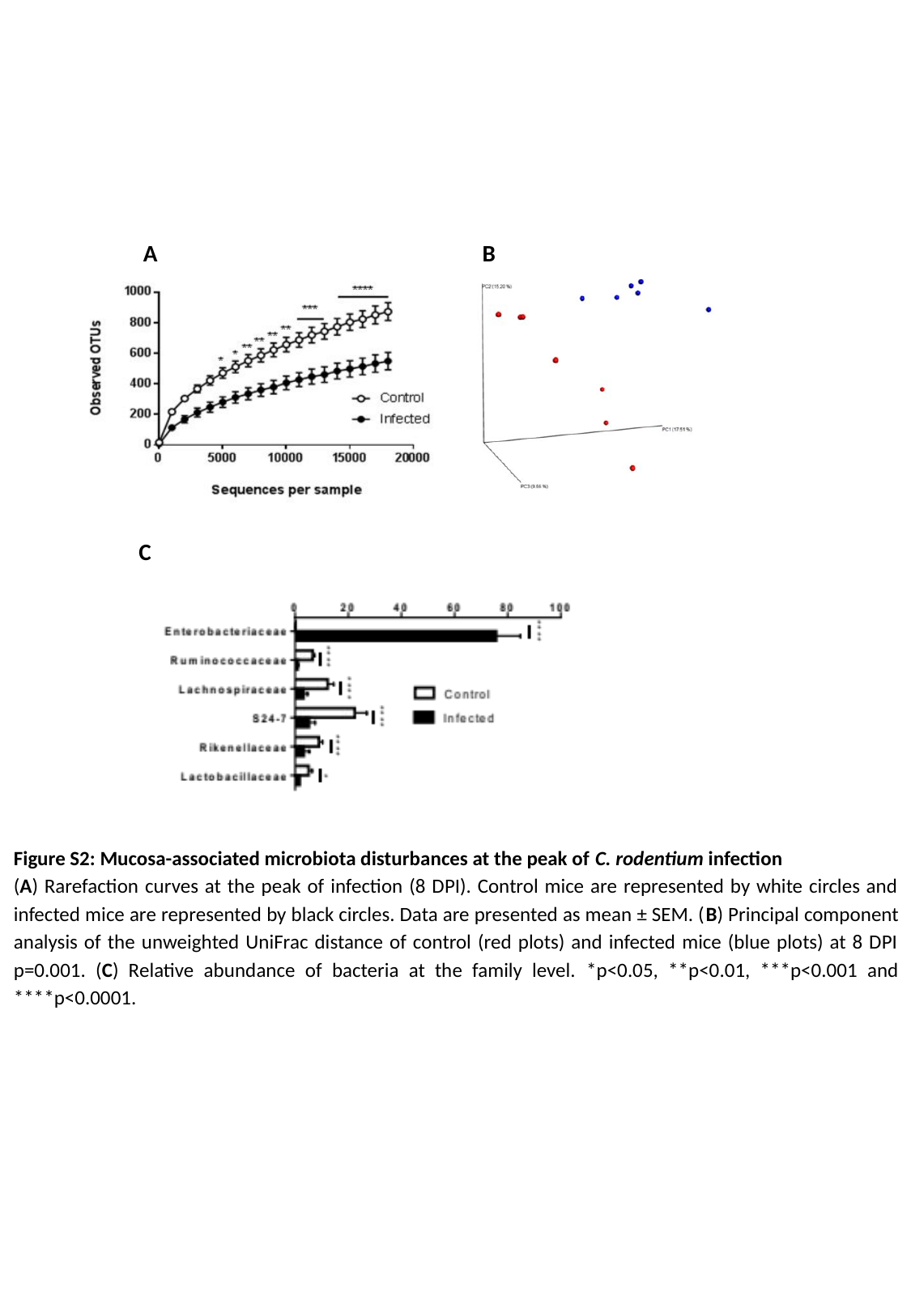

A
B
C
Figure S2: Mucosa-associated microbiota disturbances at the peak of C. rodentium infection
(A) Rarefaction curves at the peak of infection (8 DPI). Control mice are represented by white circles and infected mice are represented by black circles. Data are presented as mean ± SEM. (B) Principal component analysis of the unweighted UniFrac distance of control (red plots) and infected mice (blue plots) at 8 DPI p=0.001. (C) Relative abundance of bacteria at the family level. *p<0.05, **p<0.01, ***p<0.001 and ****p<0.0001.

## Slide 9
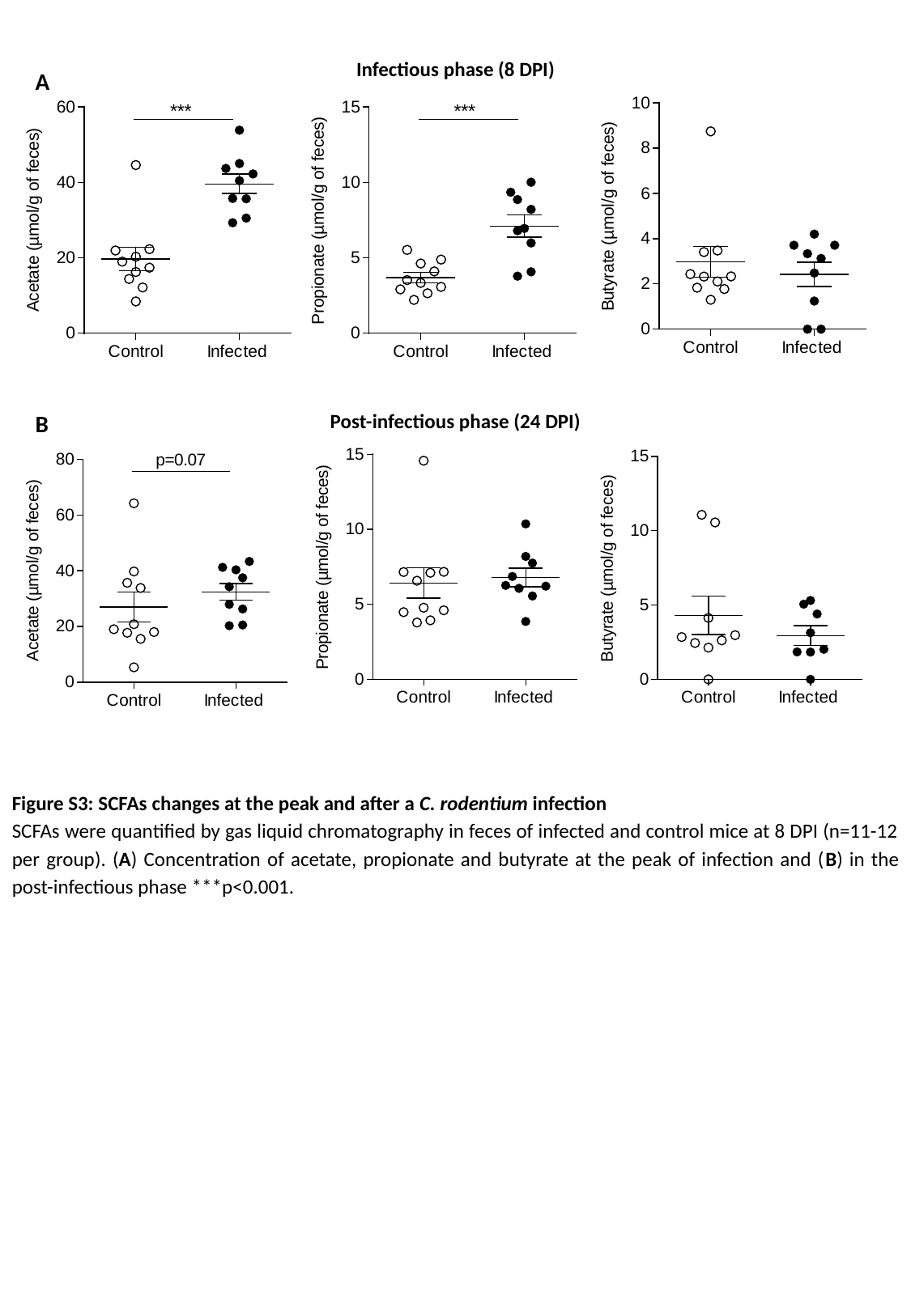

Infectious phase (8 DPI)
A
Post-infectious phase (24 DPI)
B
Figure S3: SCFAs changes at the peak and after a C. rodentium infection
SCFAs were quantified by gas liquid chromatography in feces of infected and control mice at 8 DPI (n=11-12 per group). (A) Concentration of acetate, propionate and butyrate at the peak of infection and (B) in the post-infectious phase ***p<0.001.

## Slide 10
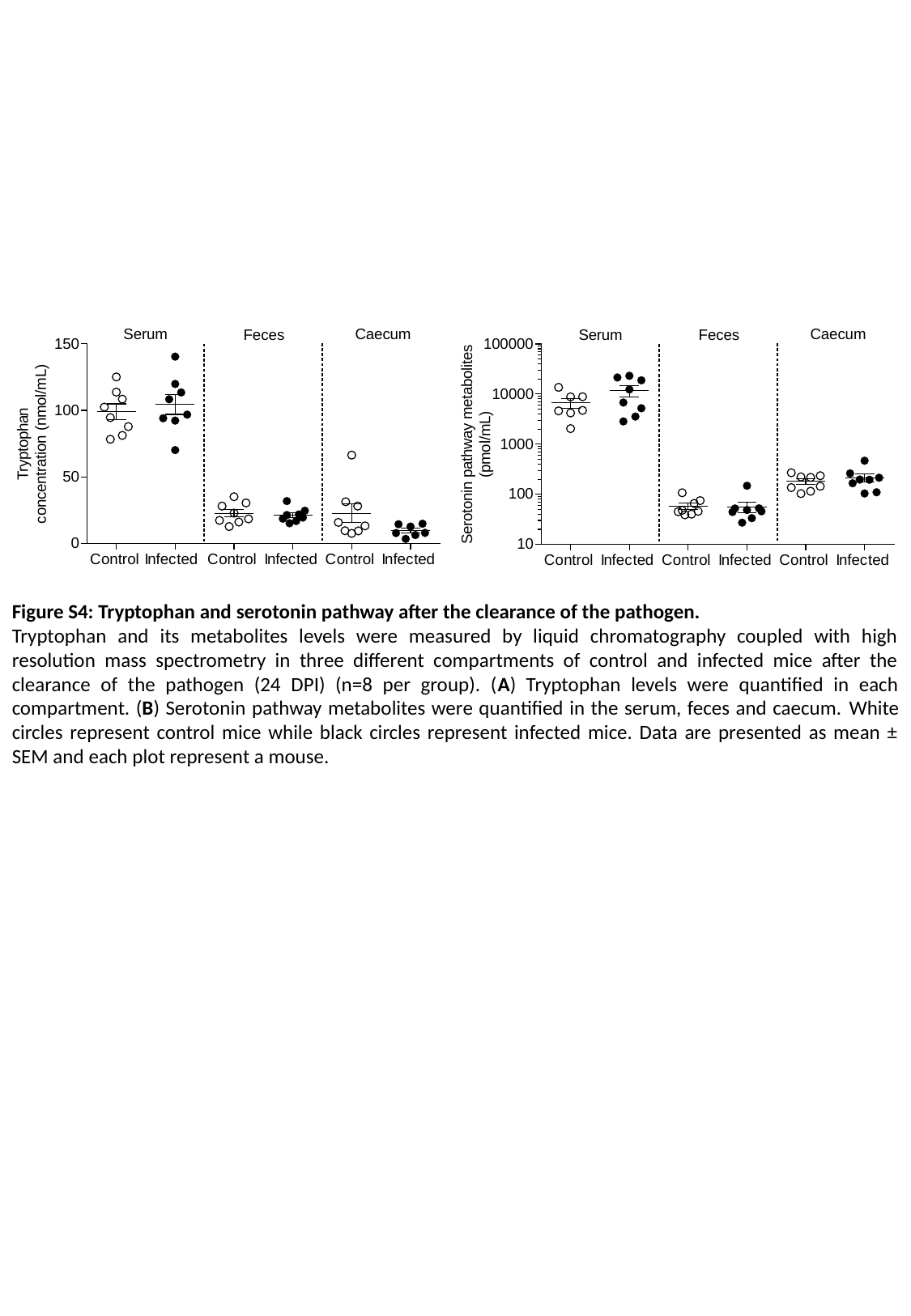

Figure S4: Tryptophan and serotonin pathway after the clearance of the pathogen.
Tryptophan and its metabolites levels were measured by liquid chromatography coupled with high resolution mass spectrometry in three different compartments of control and infected mice after the clearance of the pathogen (24 DPI) (n=8 per group). (A) Tryptophan levels were quantified in each compartment. (B) Serotonin pathway metabolites were quantified in the serum, feces and caecum. White circles represent control mice while black circles represent infected mice. Data are presented as mean ± SEM and each plot represent a mouse.

## Slide 11
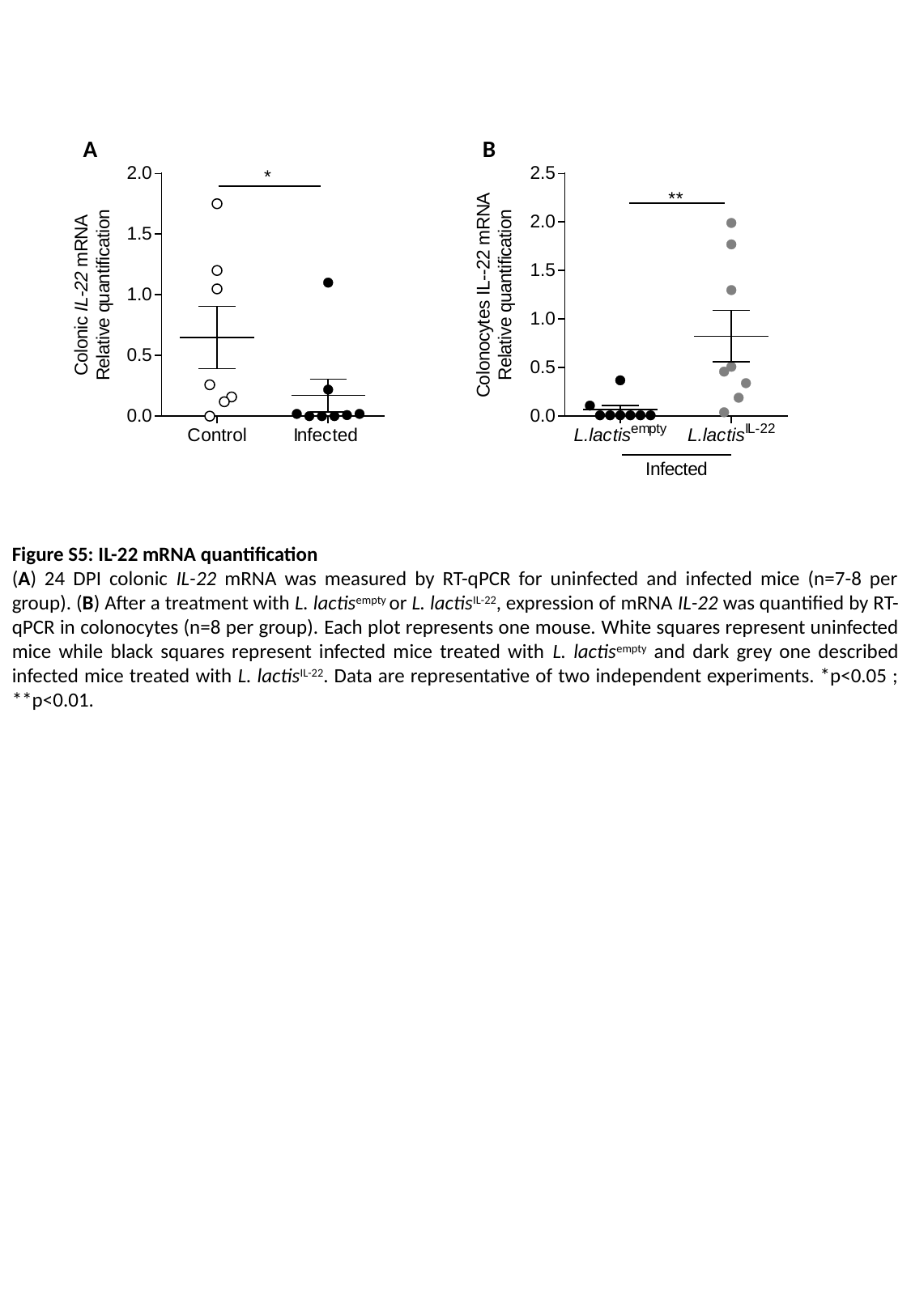

A
B
Figure S5: IL-22 mRNA quantification
(A) 24 DPI colonic IL-22 mRNA was measured by RT-qPCR for uninfected and infected mice (n=7-8 per group). (B) After a treatment with L. lactisempty or L. lactisIL-22, expression of mRNA IL-22 was quantified by RT-qPCR in colonocytes (n=8 per group). Each plot represents one mouse. White squares represent uninfected mice while black squares represent infected mice treated with L. lactisempty and dark grey one described infected mice treated with L. lactisIL-22. Data are representative of two independent experiments. *p<0.05 ; **p<0.01.

## Slide 12
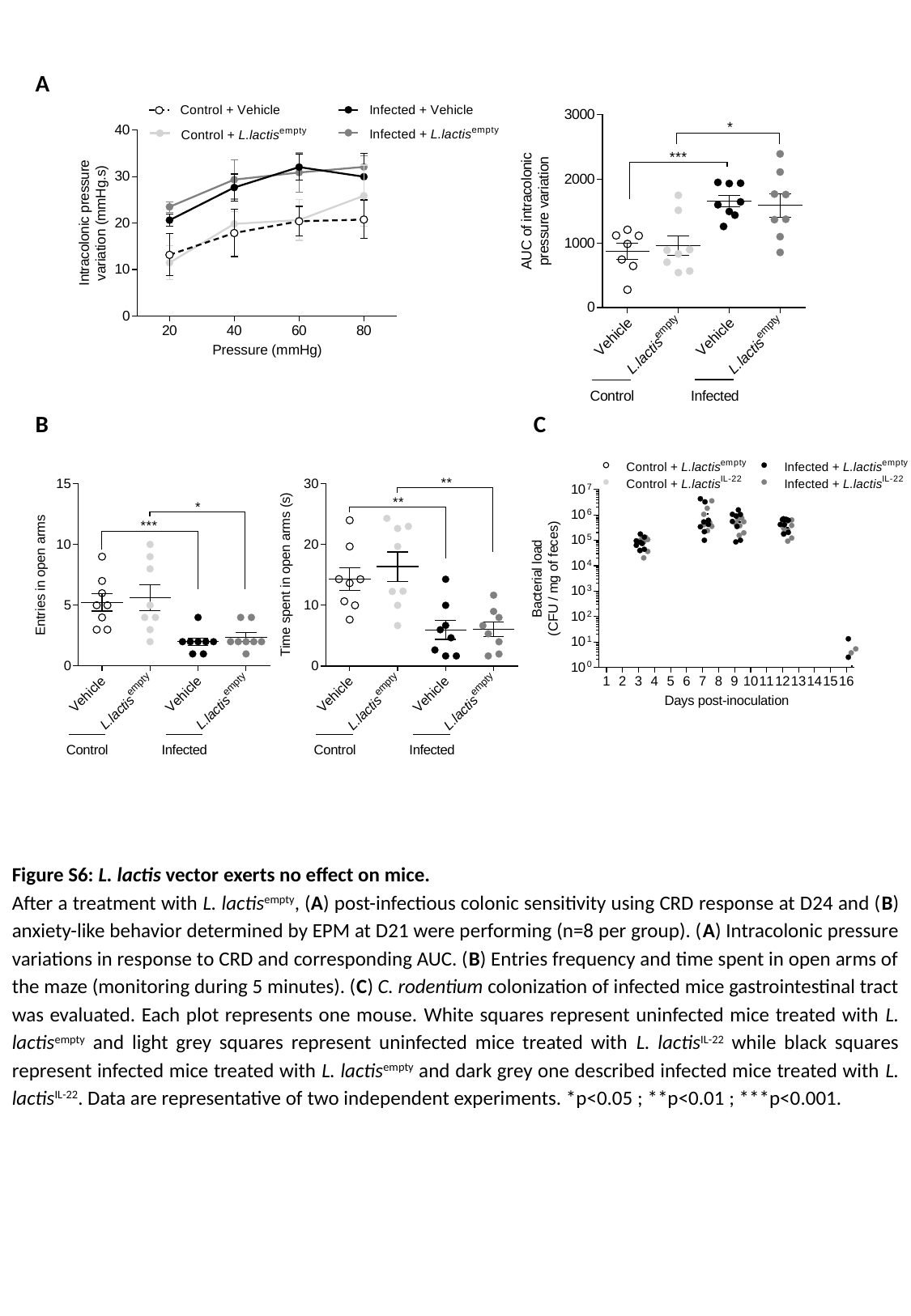

A
B
C
Figure S6: L. lactis vector exerts no effect on mice.
After a treatment with L. lactisempty, (A) post-infectious colonic sensitivity using CRD response at D24 and (B) anxiety-like behavior determined by EPM at D21 were performing (n=8 per group). (A) Intracolonic pressure variations in response to CRD and corresponding AUC. (B) Entries frequency and time spent in open arms of the maze (monitoring during 5 minutes). (C) C. rodentium colonization of infected mice gastrointestinal tract was evaluated. Each plot represents one mouse. White squares represent uninfected mice treated with L. lactisempty and light grey squares represent uninfected mice treated with L. lactisIL-22 while black squares represent infected mice treated with L. lactisempty and dark grey one described infected mice treated with L. lactisIL-22. Data are representative of two independent experiments. *p<0.05 ; **p<0.01 ; ***p<0.001.

## Slide 13
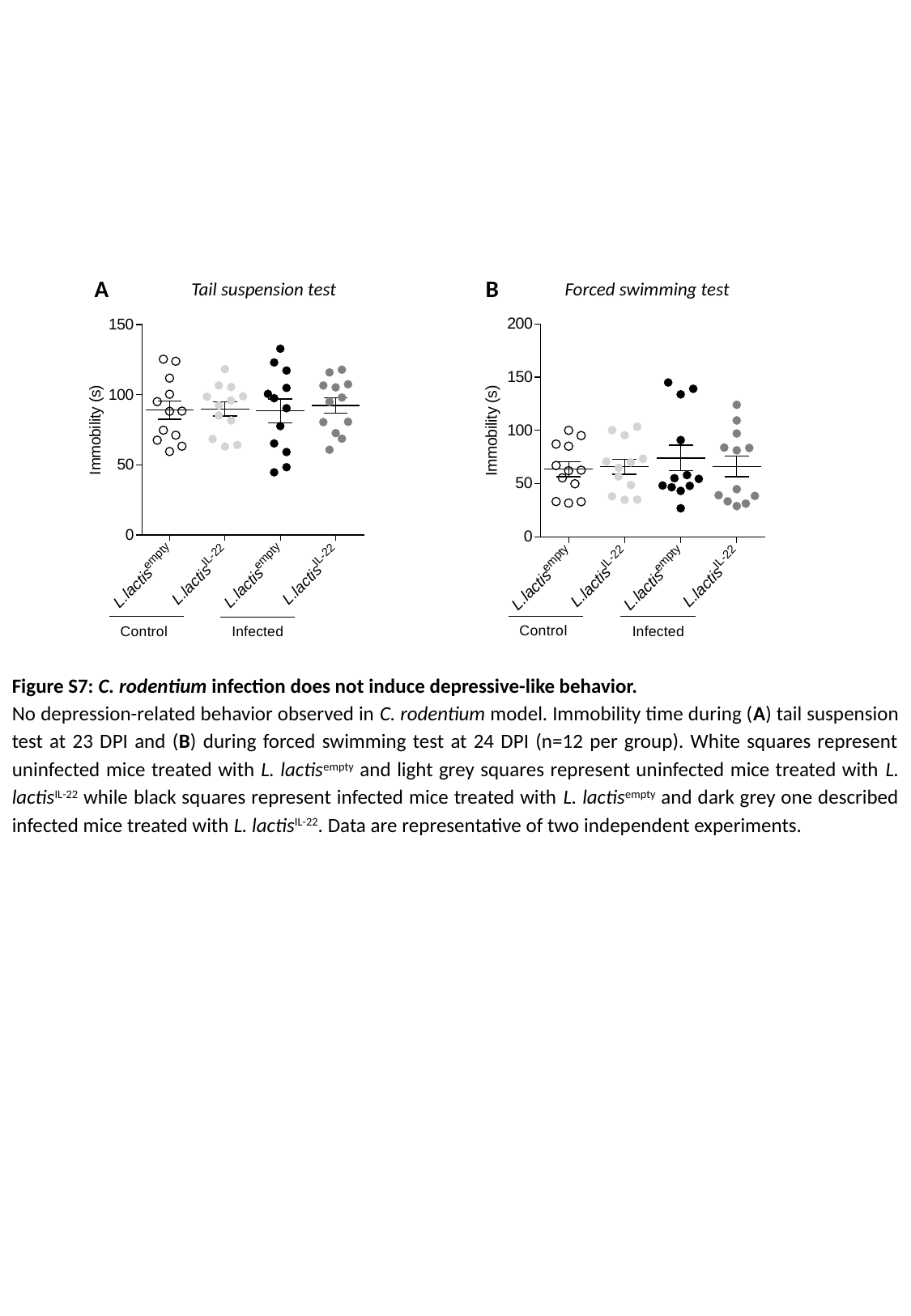

B
A
Tail suspension test
Forced swimming test
Figure S7: C. rodentium infection does not induce depressive-like behavior.
No depression-related behavior observed in C. rodentium model. Immobility time during (A) tail suspension test at 23 DPI and (B) during forced swimming test at 24 DPI (n=12 per group). White squares represent uninfected mice treated with L. lactisempty and light grey squares represent uninfected mice treated with L. lactisIL-22 while black squares represent infected mice treated with L. lactisempty and dark grey one described infected mice treated with L. lactisIL-22. Data are representative of two independent experiments.
